# Synthesis and Biological Evaluation of Novel Indole-Derived Thioureas

**DOI:** 10.3390/molecules23102554

**Published:** 2018-10-07

**Authors:** Giuseppina Sanna, Silvia Madeddu, Gabriele Giliberti, Sandra Piras, Marta Struga, Małgorzata Wrzosek, Grażyna Kubiak-Tomaszewska, Anna E. Koziol, Oleksandra Savchenko, Tadeusz Lis, Joanna Stefanska, Piotr Tomaszewski, Michał Skrzycki, Daniel Szulczyk

**Affiliations:** 1 Department of Biomedical Science, University of Cagliari, 09042 Monserrato, Italy; g.sanna@unica.it (G.S.); silvia.madeddu@unica.it (S.M.); 2 Department of Chemistry and Pharmacy, University of Sassari, 07100 Sassari, Italy; piras@uniss.it; 3 Chair and Department of Biochemistry, First Faculty of Medicine, Medical University, 02-097 Warszawa, Poland; mstruga@wum.edu.pl (M.S.); daniel.szulczyk@wum.edu.pl (D.S.); 4 Laboratory of Centre for Preclinical Research, Medical University of Warsaw, 02-097 Warsaw, Poland; malgorzata.wrzosek@wum.edu.pl; 5 Department of Biochemistry and Pharmacogenomics, Faculty of Pharmacy, Medical University of Warsaw, 02-097 Warsaw, Poland; grazyna.kubiak-tomaszewska@wum.edu.pl; 6 Faculty of Chemistry, Maria Curie-Sklodowska University, 20-031 Lublin, Poland; anna.koziol@poczta.umcs.lublin.pl (A.E.K.); oleksandra.savchenko@poczta.umcs.lublin.pl (O.S.); 7 Faculty of Chemistry, Univeristy of Wrocław, 50-383 Wrocław, Poland; tadeusz.lis@chem.uni.wroc.pl; 8 Department of Pharmaceutical Microbiology, Medical University, 02-007 Warszawa, Poland; joanna.stefanska@wum.edu.pl; 9 Department of Biochemistry, Second Faculty of Medicine, Medical University of Warsaw, 02-097 Warszawa, Poland; piotr.tomaszewski@wum.edu.pl (P.T.); mskrzycki@wum.edu.pl (M.S.)

**Keywords:** antibacterial activity, anti-HIV activity, antiviral activity, thiourea derivatives of indole, topoisomerase

## Abstract

A series of 2-(1*H*-indol-3-yl)ethylthiourea derivatives were prepared by condensation of 2-(1*H*-indol-3-yl)ethanamine with appropriate aryl/alkylisothiocyanates in anhydrous media. The structures of the newly synthesized compounds were confirmed by spectroscopic analysis and the molecular structures of **8** and **28** were confirmed by X-ray crystallography. All obtained compounds were tested for antimicrobial activity against Gram-positive cocci, Gram-negative rods and for antifungal activity. Microbiological evaluation was carried out over 20 standard strains and 30 hospital strains. Compound **6** showed significant inhibition against Gram-positive cocci and had inhibitory effect on the *S. aureus* topoisomerase IV decatenation activity and *S. aureus* DNA gyrase supercoiling activity. Compounds were tested for cytotoxicity and antiviral activity against a large panel of DNA and RNA viruses, including HIV-1 and other several important human pathogens. Interestingly, derivative **8** showed potent activity against HIV-1 wild type and variants bearing clinically relevant mutations. Newly synthesized tryptamine derivatives showed also a wide spectrum activity, proving to be active against positive- and negative-sense RNA viruses.

## 1. Introduction

**Figure 1:**
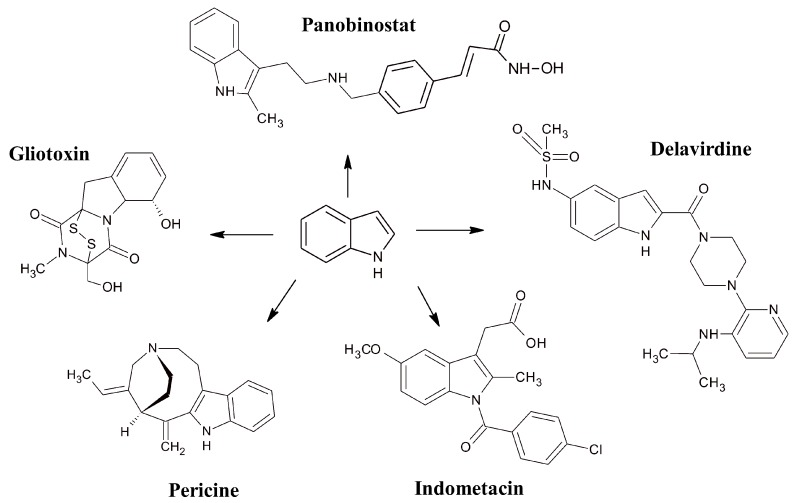
Pharmacologically active indole ring-containing derivatives.

Indole derivatives have attracted a great deal of attention due to their wide therapeutic applications (Figure 1). Tryptamine-containing arylpiperazine derivatives (e.g., delavirdine, ateviridine) belong to the group of non-nucleoside reverse transcriptase inhibitors (NNRTIs), used in the treatment of AIDS that represents the major health problem worldwide, with 1.0 million deaths and 36.7 million people living with HIV in 2016 [1]. Anti-retroviral therapy, nowadays based on combinations of drugs belonging to the NNRTI, nucleoside reverse transcriptase (NRTI), protease (PR) and, more recently, integrase (IN) inhibitor classes, has improved the quality of life of AIDS patients. However, due to a side effect and rapid emergence of drug-resistant viruses drug [2], a continuous development of new anti-HIV compounds is needed to overcome these limitations.

Indole-derived azonine compounds, such as pericine, are CNS-active alkaloids, ligands of opioid receptors. Gliotoxin and its analogues act as antimicrobials and have antiproliferative activity in lymphosarcoma cells. Indometacin is a non-steroidal anti-inflammatory agent that reduces fever, pain or swelling. Current research on panobinostat is focused on its antiproliferative effects in various cancer cell lines [3].

In order to search for potent inhibitors of hepatitis B virus (HBV) and hepatitis C virus (HCV), a series of N-substituted tryptamine analogues were synthesized [4,5]. What is more, series of 1,3,5-triazine derivatives of tryptamine show anti-HIV activity both by inhibiting cell-free virus infection and cell-to-cell virus transmission [6]. Furthermore, various families of indole-derived compounds exhibit antimicrobial activity against Gram-positive [7,8,9,10,11] and Gram-negative bacteria [7,9,10], as well as fungi [7,12]. The cytotoxic [13,14,15,16] and anticancer potency of substituted indole compounds was also proven. In this field they act as inhibitors of serine/threonine kinase (AKT) [17], tyrosinase [18], human sirtuins SIRT1-3 [19] or transcription factors NF-kB activation [20].

The thiourea moiety is an important synthon responsible for numerous biological activities, such as antimicrobial [21,22,23] antiviral [24,25], antiproliferative [26] and cytotoxic effects [22,27]. The 1,3-disubstituted thiourea scaffold is a structure with a huge potential for multi-direction activity. Through rationally planned modifications of thiourea derivative structures, a wide range of compounds with high pharmacological activity and low side-effects can be obtained. The literature indicates that including an electron acceptor group (e.g., a halogen atom) in a strategic part of a molecule significantly influences its biological activity, leading to an increase in lipophilicity, and as a result in the speed of absorption and transport of substances in vivo. The presence of a strongly electronegative substituent also changes the properties of the adjacent functional groups, which influences the reactivity, metabolic stability and chemical nature of the molecule [28,29,30]. High stability, in turn, increases the bioavailability of the compound, which improves its pharmacokinetic parameters. For these reasons, halogen derivatives constitute ca. 20% of the total number of medical drugs currently available on the world market [29]. Encouraged by the above observations and considering the interesting pharmacological profile of indole derivatives, we have synthesized new tryptamines incorporating the 1,3-disubstituted thiourea scaffold as promising antimicrobial and antiviral agents.

## 2. Results and Discussion

### 2.1. Chemistry

**Scheme 1:**
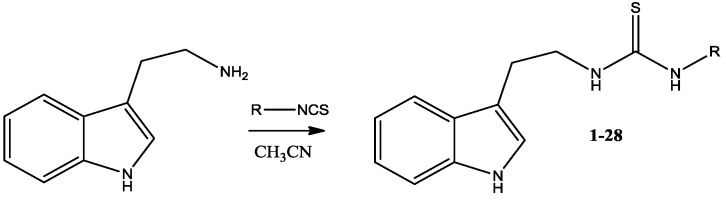
Synthesis of thiourea derivatives of 2-(1*H*-indol-3-yl)ethanamine.

The synthesis of 28 thiourea derivatives is described. Sixteen of them had not been described previously in the literature. To prepare the thiourea derivatives 2-(1*H*-indol-3-yl)ethanamine was condensed with the corresponding isothiocyanate (Scheme 1 and Table 1).

Obtained compounds were purified by column chromatography and/or crystallized from a nonpolar solvent. MS, ^1^H-NMR and ^13^C-NMR spectra confirmed the identity of the products. The molecular structures of **8** and **28** (Supplementary Material Figure S1) were also determined by an X-ray crystal structure analysis. 

### 2.2. Microbiology

Twenty eight thiourea derivatives of tryptamine were examined in vitro against Gram-positive cocci, Gram-negative rods and *Candida albicans*. The microorganisms used in this study have universal utility in the antimicrobial tests in the research for new active agents [21]. All tested compounds were screened by disc diffusion method [31], next these showing significant activity, were examined for their minimal inhibitory concentration (MIC) [32]. The results of antimicrobial activity are summarized in Table 2. In Table 2 only the results for active derivatives are included.

Preliminary tests by disc-diffusion method indicated antimicrobial activity against standard Gram-positive cocci. The next step was estimation of MIC values for not only standard but also hospital strains. The study was carried out over 20 standard, 20 hospital of *Staphylococcus aureus* and 10 hospital of *Staphylococcus epidermidis* strains used for routine antimicrobial media susceptibility testing. Hospital strains were isolated from different biological materials of the patients hospitalized in the Warsaw Medical University hospitals. MIC values for the standard Gram-positive strains were in the range 400–6.25 μg/mL (Table 3). For the hospital *Staphylococcus epidermidis* (MSSA) rods the MIC value ranged from 400 to 6.25 μg/mL and the average value was 25 μg/mL.

On the other hand, the MIC values obtained for the hospital *Staphylococcus aureus* (MSSA) for the derivatives **6** and **14** varied in the range 25–6.25 μg/mL. MRSA strains were also susceptible to these compounds, and the MIC value range changed from 25 to 6.25 μg/mL. The comparison between the chemical structure and the biological activity led to the conclusion that thiourea-derived tryptamines with halogen substituents at the ring are more potent antimicrobials than alkylthioureas. Analysis of a position and a type of an electronegative element at a benzene ring showed that chlorine at the position C-3 promotes antimicrobial activity (derivatives **6** and **14**). Substitution of the benzene ring with a second chlorine atom in the 4-position increases antimicrobial activity (compound **6**). The introduction of another substituent in the 4-position (fluorine, methyl) causes the disappearance of antimicrobial activity (compounds **23**, **28**). Other halogens (F and Br) in position 3 do not affect antimicrobial activity. On the other hand, the substitution of the benzene ring with bromine at position 2 implies the moderate antimicrobial activity, as in the previous derivatives, only for Gram-positive strains (compound **15**). The methallyl indole derivative **19** expressed a moderate antibacterial activity. These results are in the general agreement with our previous research concerning the activity of heterocyclic thiourea compounds [33]. To assess the mechanism of antimicrobial activity, *S. aureus* topoisomerase IV decatenation assay and *S. aureus* DNA gyrase supercoiling assay were performed. Ciprofloxacin (CFX) was used as a positive control. The decatenation activity of *S. aureus* topoisomerase IV was almost completely inhibited by CFX at 32 µg/mL and compound **6** has an inhibitory activity against *S. aureus* topoisomerase with IC_50_ = 28.9 ± 0.3 µg/mL (Table 4). Compound **6** shows inhibition of the supercoiling activity of *S. aureus* gyrase but weaker than that of *S. aureus* topoisomerase, and the IC_50_ for inhibition of DNA gyrase supercoiling activity for compound **6** was 72.6 ± 1.2 µg/mL (Table 4). 

**Figure 2:**
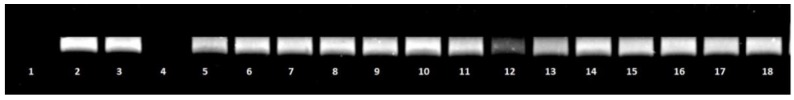
Effect of the tested compounds on *S. aureus* topoisomerase IV activity. Decreasing amounts of compounds **14** and **6** were incubated with 200 ng kinetoplast DNA and run on agarose gel. Lane 1: incubation mixture without enzyme (negative control). Lane 2: *S. aureus* topoisomerase IV assay with dilution buffer (control). Lane 3: *S. aureus* topoisomerase IV with DMSO (control). Lane: 4: Ciprofloxacin at concentration 32 μg/mL. Lane: 5–11: compound **14** at concentrations 64, 32, 16, 8, 4, 2, 0.5 μg/mL, respectively. Lane: 12–18: compound **6** at concentrations 64, 32, 16, 8, 4, 2, 0.5 μg/mL, respectively.

On the contrary, compound **14** was not significantly effective against DNA topoisomerase IV (Figure 2) and DNA gyrase (data not shown).

### 2.3. Antiviral and Antiproliferative Evaluation

All twenty-eight thiourea derivatives were tested in cell-based assays against the Human Immunodeficiency Virus type-1 (HIV-1) and against representative of several RNA and DNA virus families. The results are reported in the Table 5 and Table 6.

Interestingly, fourteen derivatives exhibited an antiviral activity, although in some cases not very potent, against one or more viruses. None of them turned out to be active against YFV, Sb-1, VSV, VV and HSV-1; also the activity against Reo-1 of derivative **18** was not particularly significant. In general, tryptamine derivatives showed different degree of cytotoxicity for the cell monolayers used to support the multiplication of different viruses (MDBK, BHK, Vero-76) in stationary growth; in particular, sixteen compounds turned out to be cytotoxic against all cell lines used, four compounds turned out to be not cytotoxic.

Only derivative **8** showed interesting activity (EC_50_ = 8.7 μM) against HIV-1, associated with a moderate cytotoxicity (CC_50_ = 45 µM). As far as the antiviral activity is concerned, six compounds (**5**, **7**, **16**, **20**, **26**, **28**) showed activity against BVDV, a member of *Pestiviruses* that have a serious impact on livestock [34], associated in two cases (**16**, **20**) with lack of cytotoxicity for MDBK cell lines. Five compounds (**10**, **13**, **14**, **16, 28**) exhibited activity (EC_50_ from 9.0 up to 30 μM) against CV-B5 virus, a member of *Enterovirus* genus that are important human pathogens that cause both acute and chronic diseases in infants, young children and immunocompromised individuals [35]. Finally, three derivatives (**3**, **15**, **16**) resulted moderately active against RSV, the most common respiratory pathogen in infants and young children worldwide [36].

Since a critical issue in the long-term clinical management of HIV disease is the development of drug resistance, compound **8** was further tested against a set of viruses possessing mutations that confer selective resistance to NRTI and NNRTI inhibitors, and that often appear during HAART therapy, reducing its effectiveness [37,38]. Interestingly, the activity against A17, AZT^R^ and MDR strains (Table 7) is comparable with those of HIV-1 wild-type, demonstrating its activity against variants carrying clinically relevant NRTI and NNRTI mutations.

Generally, no correlation could be found between the type of substituent in position 3 of the thiourea and antiviral activity. It can only be seen that aliphatic substituents (cyclohexyl, ethyl, methyl) block both antimicrobial and antiviral activity (compounds **9**, **22**, **25**). Other derivatives, those with an aromatic ring or a double bond (allyl, methallyl) are characterized by activity against various viruses. Many of the tested compounds showed a moderate cytotoxicity against the MT4 cells, with the compound **21** interestingly turning out to be cytotoxic at a very low value (CC_50_ = 2.4 µM). Its antiproliferative properties were further evaluated against different cell lines derived from human haematological tumours (Table 8), showing potent activities at micromolar level (CC_50_ = 5.0–12 µM) and confirming its promising potential.

Biological data suggested that tryptamine derivatives proved to be active against different viruses. Their wide spectrum of activity against different RNA viruses and their different degree of cytotoxicity offer interesting indications for SAR studies, with the aim to design and develop more potent derivatives. Of particular interest are the derivative **8**, strongly active against HIV-1 and variants carrying clinically relevant NRTI and NNRTI mutations, and the derivative **16** that showed activity, although moderate, against three different RNA viruses, belonging to both ssRNA+ (BVDV, CV-B5) and ssRNA-(RSV), with lack of cytotoxicity (CC_50_ > 100 µM) for MDBK and Vero-76 cell lines.

## 3. Materials and Methods

### 3.1. General Information

^1^H-NMR and ^13^C-NMR spectra were recorded on a model Avance DMX 300 spectrometer (Bruker, Billerica, MA, USA, ^1^H at 300 MHz and ^13^C at 75, MHz respectively). The chemical shift values are expressed in ppm relative to TMS as an internal standard. Mass spectral ESI measurements were carried out on a ZQ Micromass instrument (Waters, Milford, MA, USA) equipped with a quadrupole mass analyzer. The spectra were recorded in the positive ion mode at a declustering potential of 40–60 V. The samples were previously separated on a UPLC column (C18) using an ACQUITY UPLC system by Waters connected with a DPA detector. Flash chromatography was performed on silica gel 60 (200–400 mesh, Merck, Kenilworth, NJ, USA) using chloroform/methanol (19:1 vol) mixture as eluent. Analytical TLC was carried out on silica gel F254 (Merck) plates (0.25 mm thickness). 

The diffraction data for the X-ray crystal structure analysis were collected for **8** and **28** at 200 K and 150 K, respectively, with an Xcalibur CCD diffractometer (Oxford Diffraction Ltd, Abingdon, UK), using graphite monochromated MoKα radiation. Crystal structures were solved by direct methods using the SHELXS-97 program and refined by full-matrix least squares method on *F*^2^ using the SHELXL-97 program [39]. All ordered non-hydrogen atoms were refined with anisotropic displacement parameters. Hydrogen atoms were positioned geometrically and allowed to ride on their parent atoms, with Uiso(H) = 1.2 Ueq(C). The experimental details and final atomic parameters for **8** and **28** have been deposited with the Cambridge Crystallographic Data Centre as supplementary material (CCDC ID: 992,428 and 992,429). Copies of the data can be obtained free of charge on request by e-mailing data_request@ccdccam.ac.uk or via www.ccdc.cam.ac.uk/data_request/cif.

### 3.2. Chemistry

#### General Procedure for the Synthesis of Thiourea Derivatives of 2-(1H-indol-3-yl)ethanamine

A solution of 2-(1*H*-indol-3-yl)ethanamine (0.0038 mol, 0.61 g) in anhydrous acetonitrile (25 mL) was treated with an appropriate isothiocyanate (0.0042 mol) and the mixture was refluxed for 8 h. Then solvent was removed on rotary evaporator. The residue was purified by column chromatography (chloroform/methanol; 9.8:0.2 vol.). The compound was crystallized from acetonitrile or another appropriate solvent.

*1-(2-(1H-Indol-3-yl)ethyl)-3-phenylthiourea* (**1**). This compound was synthesized as described previously [40].

*1-(2-(1H-Indol-3-yl)ethyl)-3-(4-methoxyphenyl)thiourea* (**2**). Yield 87%. m.p. 146–148 °C. ^1^H-NMR (DMSO-*d*_6_) δ (ppm): 3.07 (t, 2H, CH_2_, *J* = 6.6 Hz); 3.77 (s, 3H, CH_3_); 3.89 (t, 2H, CH_2_, *J* = 6.3 Hz); 6.42 (s, 1H, NH); 6.71 (d, 1H,CHarom., *J* = 8.7 Hz); 6.83 (d, 2H, CH_arom_., *J* = 9.3 Hz); 7.02 (bs, 1H, NH); 7.35–7.41 (m, 3H, CH_arom_.); 7.55–7.67 (m, 3H, CH_arom_.); 7.99 (bs, 1H, NH). ^13^C-NMR (DMSO-*d*_6_) δ (ppm): 24.6 (CH_2_), 44.6 (CH_2_), 55.2 (CH_3_), 111.3 (C), 111.6 (CH), 113.9 (CH, CH), 118.2 (CH), 118.5 (CH), 120.9 (CH), 122.7 (CH), 125.8 (CH), 127.3 (C), 128.6 (C), 131.5 (C), 136.3 (C), 156.5 (CH), 180.4 (C). HRMS (ESI) calc. for C_18_H_19_N_3_OS [M − H]^−^: 324.4279, found: 324.4298.

*1-(2-(1H-Indol-3-yl)ethyl)-3-(4-methylphenyl)thiourea* (**3**). This compound was synthesized as described previously [41].

*1-(2-(1H-Indol-3-yl)ethyl)-3-(4-chlorophenyl)thiourea* (**4**). This compound was synthesized as described previously [39].

*1-(2-(1H-Indol-3-yl)ethyl)-3-benzoylthiourea* (**5**). This compound was synthesized as described previously [42].

*1-(2-(1H-indol-3-yl)ethyl)-3-(3,4-dichlorophenyl)thiourea* (**6**). Yield 81%. m.p. 168–169 °C. ^1^H-NMR (DMSO-*d*_6_) δ (ppm): 2.99 (t, 2H, CH_2_, *J* = 7.2 Hz); 3.76 (q, 2H, CH_2_, *J* = 5.4 Hz); 6.98 (t, 1H, CH_arom_., *J* = 7.8 Hz); 7.08 (t, 1H,CH_arom_., *J* = 7.2 Hz); 7.18 (d, 1H, CH_arom_., *J* = 2.4 Hz); 7.30–7.36 (m, 2H, CH_arom_.); 7.52 (d, 1H,CH_arom_., *J* = 8.7 Hz); 7.62 (d, 1H, CH_arom_., *J* = 7.8 Hz); 7.85 (s, 1H, CH_arom_.); 7.99 (bs, 1H, NH); 9.72 (s, 1H, NH); 10.84 (s, 1H, NH). ^13^C-NMR (DMSO-*d*_6_) δ (ppm): 24.3 (CH_2_), 44.5 (CH_2_), 111.4 (C), 111.4 (CH), 118.3 (CH), 118.4 (CH), 120.9 (CH), 122.5 (CH), 122.8 (CH), 123.7 (CH), 125.3 (C), 127.2 (C), 130.2 (C), 130.5 (C), 136.3 (C), 139.7 (CH), 180.2 (C). HRMS (ESI) calc. for C_17_H_15_ Cl_2_N_3_S [M − H]^−^: 363.2921, found: 363.2943.

*1-(2-(1H-Indol-3-yl)ethyl)-3-(3-bromophenyl)thiourea* (**7**). Yield 79%. m.p. 152–154 °C. ^1^H-NMR (DMSO-*d*_6_) δ (ppm): 3.09 (t, 2H, CH_2_, *J* = 6.6 Hz); 3.97 (q, 2H, CH_2_, *J* = 5.7 Hz); 6.06 (s, 1H, NH); 6.81 (d, 1H, CH_arom_., *J* = 7.2 Hz); 7.00 (s, 1H, CH_arom_.); 7.05 (d, 1H, CH_arom_., *J* = 8.1 Hz); 7.08 (d, 1H, CH_arom_., *J* = 9.0 Hz); 7.13 (s, 1H, CH_arom_.); 7.20 (t, 1H, CH_arom_., *J* = 7.2 Hz); 7.29 (d, 1H,CH_arom_., *J* = 8.1 Hz); 7.36 (d, 1H, CH_arom_., *J* = 8.1 Hz); 7.40 (bs, 1H, NH); 7.57–7.60 (d, 1H, CH_arom_., *J* = 8.7 Hz); 8.03 (s, 1H, NH). ^13^C-NMR (DMSO-*d*_6_) δ (ppm): 24.3 (CH_2_), 44.5 (CH_2_), 111.4 (C), 111.5 (CH), 118.3 (CH), 118.5 (CH), 121.0 (CH), 121.1 (CH), 121.3 (CH), 122.8 (CH), 124.9 (CH), 126.3 (C), 127.2 (C), 130.4 (C), 136.3 (C), 141.2 (CH), 180.1 (C). HRMS (ESI) calc. for C_17_H_16_BrN_3_S [M − H]^−^: 373.2980, found: 373.2978.

*1-(2-(1H-Indol-3-yl)ethyl)-3-(4-bromophenyl)thiourea* (**8**). Yield 81%. m.p. 156–158 °C. ^1^H-NMR (DMSO-*d*_6_) δ (ppm): 3.09 (t, 2H, CH_2_, *J* = 6.6 Hz); 3.95 (t, 2H, CH_2_, *J* = 6.6 Hz); 6.24 (bs, 1H, NH); 6.71 (d, 1H, CH_arom_., *J* = 8.7 Hz); 6.97 (s, 1H, CH_arom_.); 7.11 (t, 1H, CH_arom_., *J* = 7.8 Hz); 7.17–7.29 (m, 3H, CH_arom_.); 7.39 (d, 1H,CH_arom_., *J* = 8.1 Hz); 7.56 (d, 1H, CH_arom_., *J* = 8.1 Hz); 7.62 (bs, 1H, NH); 8.01 (bs, 1H, NH). ^13^C-NMR (DMSO-*d*_6_) δ (ppm): 24.4 (CH_2_), 44.5 (CH_2_), 111.4 (C), 111.5 (CH), 115.8 (CH), 118.2 (CH, CH), 118.5 (CH), 121.0 (CH), 122.8(CH), 124.7 (CH), 127.2 (CH), 131.3(C, C), 136.3 (C), 138.7, 180.1 (C). HRMS (ESI) calc. for C_17_H_16_BrN_3_S [M − H]^−^: 373.2980, found: 373.2978. *Crystal data:* crystal system monoclinic, space group *Cc*, unit cell dimensions *a* = 35.17(2), *b* = 10.863(4), *c* = 8.798(3) Å, β = 100.44(5) °, *V* = 3306(3) Å^3^; *Z* = 8, d_c_ = 1.504 g/cm^3^, μ = 2.613 mm^−1^, *F*(000) = 1520. A crystal of dimensions 0.20 × 0.15 × 0.04 mm was used for intensity measurements. Within the θ range 2.98–26.31° (−43 ≤ *h* ≤ 33, −13 ≤ *k* ≤ 13, −7 ≤ *l* ≤ 10), 9510 reflections were collected. The 4871 unique reflections [*R*(int) = 0.0699] were used for the refinement of 397 parameters. Final *R* indices on *F*^2^ for 2599 observed reflections [*I* > 2σ(*I*)] were: *R*_1_ = 0.0615, *wR*_2_ = 0.0825, goodness-of-fit = 0.941, Δρ^max/min^ = 0.57/−0.44 e Å^−3^.

*1-(2-(1H-Indol-3-yl)ethyl)-3-cyclohexylthiourea* (**9**). This compound was synthesized as described previously [40].

*1-(2-(1H-Indol-3-yl)ethyl)-3-benzylthiourea* (**10**). This compound was synthesized as described previously [43].

*1-(2-(1H-Indol-3-yl)ethyl)-3-(2-fluorophenyl)thiourea* (**11**). Yield 90%. m.p. 167–168 °C. ^1^H-NMR (DMSO-*d*_6_) δ (ppm): 3.09 (t, 2H, CH_2_, *J* = 6.6 Hz); 3.98(t, 2H, CH_2_, *J* = 6.3 Hz); 6.06 (bs, 1H, NH); 6.88–6.98 (m, 3H, CH_arom_.); 7.04–7.17 (m, 3H, CH_arom_.); 7.35 (d, 2H,CH_arom_., *J* = 8.1 Hz); 7.58 (d, 1H, CH_arom_., *J* = 7.5 Hz); 7.62 (bs, 1H, NH); 7.99 (s, 1H, NH). ^13^C-NMR (DMSO-*d*_6_) δ (ppm): 24.5 (CH_2_), 44.7 (CH_2_), 111.3 (C), 111.5 (CH), 115.6 (CH), 115.9 (d, *J* = 31.2 Hz, CH), 118.2 (CH), 118.5 (CH), 121.0 (CH), 122.8 (CH), 124.0 (d, *J* = 3.7 Hz, CH), 126.6 (d, *J* = 11.8 Hz, CH), 127.2 (C), 127.9 (d, *J* = 7.8 Hz, C), 136.3 (C), 157.5 (d, *J* = 245.3Hz, CH), 181.1 (C). HRMS (ESI) calc. for C_17_H_16_FN_3_S [M − H]^−^: 312.3924, found: 312.3921.

*1-(2-(1H-Indol-3-yl)ethyl)-3-(3-fluorophenyl)thiourea* (**12**). Yield 88%. m.p. 162–164 °C. ^1^H-NMR (DMSO-*d*_6_) δ (ppm): 3.10 (t, 2H, CH_2_, *J* = 6.6 Hz); 3.98 (q, 2H, CH_2_, *J* = 5.7 Hz); 6.13 (s, 1H, NH); 6.65 (d, 2H, CHarom., *J* = 6.9 Hz); 6.87 (d, 1H, CH_arom_., *J* = 6.9 Hz); 6.98 (s, 1H, CH_arom_.); 7.05–7.23 (m, 3H, CH_arom_.); 7.37 (d, 2H, CH_arom_., *J* = 8.1 Hz); 7.49 (s, 1H, NH); 7.58–7.60 (d, 2H, CH_arom_., *J* = 7.8 Hz); 8.01 (s, 1H, NH). ^13^C-NMR (DMSO-*d*_6_) δ (ppm): 24.4 (CH_2_), 44.5 (CH_2_), 109.2 (C), 110.3 (d, *J* = 24,9 Hz, CH), 111.4 (d, *J* = 21,3 Hz, CH), 111.5 (CH), 118.3 (CH), 118.5 (q, *J* = 3.9 Hz, CH), 121.0 (CH), 122.8 (CH), 127.2 (CH), 130.1 (q, *J* = 32 Hz, CH), 136.3 (C), 141.3 (d, *J* = 10.8 Hz, C), 160.3 (d, *J* = 242 Hz, C), 163.5 (CH), 180.1 (C). HRMS (ESI) calc. for C_17_H_16_FN_3_S [M − H]^−^: 312.3924, found: 312.3921.

*1-(2-(1H-Indol-3-yl)ethyl)-3-(2-chlorophenyl)thiourea* (**13**). Yield 79%. m.p. 170–172 °C. ^1^H-NMR (DMSO-*d*_6_) δ (ppm): 2.97 (t, 2H, CH_2_, *J* = 7.5 Hz); 3.75 (q, 2H, CH_2_, *J* = 5.7 Hz); 6.98 (t, 1H,CHarom., *J* = 7.5 Hz); 7.07 (t, 1H, CH_arom_., *J* = 6.9 Hz); 7.16–7.35 (m, 4H, CH_arom_.); 7.47 (d, 1H,CH_arom_., *J* = 7.8 Hz); 7.63 (d, 2H, CH_arom_., *J* = 6.9 Hz); 7.95 (bs, 1H, NH); 9.16 (s, 1H, NH); 10.83 (s, 1H, NH). ^13^C-NMR (DMSO-*d*_6_) δ (ppm): 24.5 (CH_2_), 44.7 (CH_2_), 111.3 (C), 111.5 (CH), 118.2 (CH), 118.5 (CH), 120.9 (CH), 122.7 (CH), 126.9 (CH), 127.1 (CH), 127.2 (CH), 128.9 (C), 129.1 (C), 129.4(C), 136.0(C), 136.2 (CH), 181.1 (C). HRMS (ESI) calc. for C_17_H_16_ClN_3_S [M − H]^−^: 328.8470, found: 328.8467.

*1-(2-(1H-Indol-3-yl)ethyl)-3-(3-chlorophenyl)thiourea* (**14**). Yield 85%. m.p. 169–171 °C. ^1^H-NMR (DMSO-*d*_6_) δ (ppm): 2.98 (t, 2H, CH_2_, *J* = 7.5 Hz); 3.77 (q, 2H, CH_2_, *J* = 5.7 Hz); 6.98 (t, 1H, CH_arom_., *J* = 8.1 Hz); 7.04–7.14 (m, 2H, CH_arom_.); 7.18 (d, 1H, CH_arom_., *J* = 2.4 Hz); 7.23–7.36 (m, 3H, CH_arom_.); 7.63 (d, 2H, CH_arom_., *J* = 8.4 Hz); 7.91 (bs, 1H, NH); 9.65 (s, 1H, NH); 10.84 (s, 1H, NH). ^13^C-NMR (DMSO-*d*_6_) δ (ppm): 24.4 (CH_2_), 44.5 (CH_2_), 111.4 (C), 111.5 (CH), 118.2 (CH, CH), 118.5 (CH), 120.96 (CH), 122.0 (CH), 122.8 (CH), 123.4 (CH), 127.2 (C), 130.1 (C), 132.6 (C), 136.3 (C), 140.9 (CH), 180.1 (C). HRMS (ESI) calc. for C_17_H_16_ClN_3_S [M − H]^−^: 328.8470, found: 328.8468.

*1-(2-(1H-Indol-3-yl)ethyl)-3-(2-bromophenyl)thiourea* (**15**). Yield 83%. m.p. 150–152 °C. ^1^H-NMR (DMSO-*d*_6_) δ (ppm): 2.98 (t, 2H, CH_2_, *J* = 7.2 Hz); 3.75 (q, 2H, CH_2_, *J* = 5.4 Hz); 6.98 (t, 1H,CH_arom_., *J* = 7.8 Hz); 7.07 (t, 1H, CH_arom_., *J* = 6.6 Hz); 7.12–7.17 (m, 2H, CH_arom_.); 7.32–7.38 (m, 2H, CH_arom_.); 7.57 (d, 1H, CH_arom_., *J* = 7.2 Hz); 7.62–7.66 (m, 2H, CH*arom*.); 7.91 (bs, 1H, NH); 9.11 (s, 1H, NH); 10.83 (s, 1H, NH). ^13^C-NMR (DMSO-*d*_6_) δ (ppm): 24.5 (CH_2_), 44.7 (CH_2_), 111.3 (C), 111.5 (CH), 118.2 (CH), 118.5 (CH), 120.2 (CH), 120.9 (CH), 122.8 (CH), 127.2 (CH), 127.4 (CH), 127.7 (C), 129.7 (C), 132.5 (C), 136.2 (C), 137.4 (CH), 181.1 (C). HRMS (ESI) calc. for C_17_H_16_BrN_3_S [M − H]^−^: 373.2980, found: 373.2978.

*1-(2-(1H-Indol-3-yl)ethyl)-3-phenethylthiourea* (**16**). This compound was synthesized as described previously [44].

*1-(2-(1H-Indol-3-yl)ethyl)-3-(4-fluorophenyl)thiourea* (**17**). This compound was synthesized as described previously [42].

*1-(2-(1H-Indol-3-yl)ethyl)-3-allylthiourea* (**18**). This compound was synthesized as described previously [40].

*1-(2-(1H-Indol-3-yl)ethyl)-3-(2-methylallyl)thiourea* (**19**). Yield 76%. m.p. 141–143 °C. ^1^H-NMR (DMSO-*d*_6_) δ (ppm): 1.67 (s, 3H, CH_3_); 2.90 (t, 2H, CH_2_, *J* = 7.2 Hz); 3.67 (bs, 2H, CH_2_); 3.98 (bs, 2H, CH_2_); 4.77 (s, 2H, =CH_2_); 6.98 (t, 1H, CH_arom_., *J* = 7.8 Hz); 7.08 (t, 1H, CH_arom_., *J* = 6.9 Hz); 7.14 (d, 1H, CH_arom_., *J* = 2.4 Hz); 7.33 (d, 1H, CH_arom_., *J* = 8.1 Hz); 7.42 (bs, 1H, NH); 7.56 (bs, 1H, NH); 7.59 (d, 1H, CH_arom_., *J* = 7.8 Hz); 10.82 (s, 1H, NH). ^13^C-NMR (DMSO-*d*_6_) δ (ppm): 20.3 (CH_3_), 24.9 (CH_2_), 44.4 (C), 48.7 (CH_2_), 109.9 (CH_2_), 111.3 (CH_2_), 111.6 (C), 118.2 (CH), 118.46 (CH), 120.9 (CH), 122.7 (CH), 127.3 (C), 136.2 (C), 142.4 (CH), 182.5 (C). HRMS (ESI) calc. for C_15_H_19_N_3_S [M − H]^−^: 272.3964, found: 272.3486.

*1-(2-(1H-Indol-3-yl)ethyl)-3-ethoxycarbonylthiourea* (**20**). This compound was synthesized as described previously [43].

*1-(2-(1H-Indol-3-yl)ethyl)-3-(4-iodophenyl)thiourea* (**21**). Yield 88%. m.p. 162–165 °C. ^1^H-NMR (DMSO-*d*_6_) δ (ppm): 2.97 (t, 2H, CH_2_, *J* = 7.5 Hz); 3.75 (q, 2H, CH_2_, *J* = 6.0 Hz); 6.98 (t, 1H, CH_arom_., *J* = 6.9 Hz); 7.07 (t, 1H,CH_arom_., *J* = 6.9 Hz); 7.20 (m, 3H, CH_arom_.); 7.34 (d, 1H, CH_arom_., *J* = 8.1 Hz); 7.61 (m, 3H, CH_arom_.); 7.83 (bs, 1H, NH); 9.57 (s, 1H, NH); 10.84 (s, 1H, NH). ^13^C-NMR (DMSO-*d*_6_) δ (ppm): 24.39 (CH_2_), 44.53 (CH_2_), 111.34 (C), 111.48 (CH), 118.22 (CH, CH), 118.46 (CH), 120.95 (CH, CH), 122.81 (CH), 124.92 (CH), 127.21 (CH), 136.26 (C), 137.13 (C, C), 139.18 (CH), 180.03 (C). HRMS (ESI) calc. for C_17_H_16_IN_3_S [M − H]^−^: 420.2985, found: 420.2987.

*1-(2-(1H-Indol-3-yl)ethyl)-3-ethylthiourea* (**22**). This compound was synthesized as described previously [45].

*1-(2-(1H-Indol-3-yl)ethyl)-3-(3-chloro-4-methylphenyl)thiourea* (**23**). Yield 83%. m.p. 161–163 °C. ^1^H-NMR (DMSO-*d*_6_) δ (ppm): 2.28 (s, 3H, CH_3_); 2.98 (t, 2H, CH_2_, *J* = 7.5 Hz); 3.76 (q, 2H, CH_2_, *J* = 5.7 Hz); 6.98 (t, 1H, CH_arom_., *J* = 8.1 Hz); 7.08 (t, 1H, CH_arom_., *J* = 6.9 Hz); 7.13–7.18 (m, 2H, CH_arom_.); 7.25 (d, 1H, CH_arom_., *J* = 8.4 Hz); 7.34 (d, 1H,CH_arom_., *J* = 7.8 Hz); 7.57 (d, 1H,CH_arom_., *J* = 2.1 Hz); 7.62 (d, 1H, CH_arom_., *J* = 7.5 Hz); 7.81 (bs, 1H, NH); 9.55 (s, 1H, NH); 10.84 (s, 1H, NH). ^13^C-NMR (DMSO-*d*_6_) δ (ppm): 18.9 (CH_3_), 24.4 (CH_2_), 44.5 (CH_2_), 111.3 (C), 111.5 (CH), 118.2 (CH), 118.5 (CH), 120.9 (CH), 121.7 (CH), 122.8 (C), 123.0 (CH), 127.2 (CH), 130.7 (C), 130.9 (C), 132.6 (C), 136.3 (C), 138.4 (CH), 180.2 (C). HRMS (ESI) calc. for C_18_H_18_ClN_3_S [M − H]^−^: 342.8736, found: 342.8769.

*1-(2-(1H-Indol-3-yl)ethyl)-3-(3-(trifluoromethyl)phenyl)thiourea* (**24**). Yield 92%. m.p. 169–171 °C. ^1^H-NMR (DMSO-*d*_6_) δ (ppm): 2.99 (t, 2H, CH_2_, *J* = 7.2 Hz); 3.79 (q, 2H, CH_2_, *J* = 5.7 Hz); 6.98 (t, 1H, CH_arom_., *J* = 7.8 Hz); 7.08 (t, 1H, CH_arom_., *J* = 7.8 Hz); 7.18 (d, 1H,CH_arom_., *J* = 2.1 Hz); 7.35 (d, 1H, CH_arom_., *J* = 8.1 Hz); 7.40 (d, 1H, CH_arom_., *J* = 7.8 Hz); 7.50 (d, 1H, CH_arom_., *J* = 7.8 Hz); 7.64 (d, 1H, CH_arom_., *J* = 7.8 Hz); 7.98 (s, 1H, CH_arom_.); 7.98 (bs, 1H, NH); 9.79 (s, 1H, NH); 10.85 (s, 1H, NH). ^13^C-NMR (DMSO-*d*_6_) δ (ppm): 24.4 (CH_2_), 44.4 (CH_2_), 79.2 (C), 111.4 (C), 111.4 (CH), 118.2 (CH), 118.4 (CH), 118.6 (CH), 121.0 (q, 3.8 Hz, CH), 122.8 (q, *J* = 3.8 Hz, CH), 125.8 (q, *J* = 272 Hz, C), 126.1 (CH), 127.2 (CH), 128.8 (C), 129.5 (q, *J* = 33.6 Hz, C), 136.3 (C), 140.4, 180.4 (C). HRMS (ESI) calc. for C_18_H_16_F_3_N_3_S [M − H]^−^: 362.3999, found: 362.3940.

*1-(2-(1H-Indol-3-yl)ethyl)-3-methylthiourea* (**25**). This compound was synthesized as described previously [46].

*1-(2-(1H-Indol-3-yl)ethyl)-3-(3-chloro-6-methylphenyl)thiourea* (**26**). Yield 87%. m.p. 163–165 °C. ^1^H-NMR (DMSO-*d*_6_) δ (ppm): 2.13 (s, 3H, CH_3_); 2.96 (t, 2H, CH_2_, *J* = 7.2 Hz); 3.73 (q, 2H, CH_2_, *J* = 5.1 Hz); 6.98 (t, 1H,CH_arom_., *J* = 6.3 Hz); 7.07 (t, 1H, CH_arom_., *J* = 6.9 Hz); 7.15–7.26 (m, 3H, CH_arom_.); 7.34 (d, 1H, CH_arom_., *J* = 8.1 Hz); 7.37 (s, 1H, CH_arom_.); 7.63 (d, 1H, CH_arom_., *J* = 7.8 Hz); 7.71 (bs, 1H, NH); 9.11 (s, 1H, NH); 10.82 (s, 1H, NH). ^13^C-NMR (DMSO-*d*_6_) δ (ppm): 17.2 (CH_3_), 24.6 (CH_2_), 44.8 (CH_2_), 111.3 (C), 111.6 (CH), 118.2 (CH), 118.5 (CH), 120.9 (CH), 122.7 (CH), 125.8 (C), 127.0 (CH), 127.3 (CH), 129.7 (C), 131.8 (C), 133.1 (C), 136.2 (C), 138.6 (CH), 180.9 (C). HRMS (ESI) calc. for C_18_H_18_ClN_3_S [M − H]^−^: 342.8736, found: 342.8772.

*1-(2-(1H-Indol-3-yl)ethyl)-3-(4-butyl-2-methylphenyl)thiourea* (**27**). Yield 77%. m.p. 172–174 °C. ^1^H-NMR (DMSO-*d*_6_) δ (ppm): 0.8 (t, 3H, CH_3_, *J* = 7.5 Hz); 1.25–1.37 (m, 2H, CH_2_, *J* = 7.5 Hz); 1.49–1.59 (m, 2H, CH_2_, *J* = 7.2 Hz); 2.11 (s, 3H, CH_3_); 2.49 (s, 2H, CH_2_); 2.93 (t, 2H, CH_2_, *J* = 7.5 Hz); 3.69 (q, 2H, CH_2_, *J* = 6.0 Hz); 6.94–7.11 (m, 6H, CH_arom_.); 7.25 (bs, 1H, NH); 7.33 (d, 1H, CH_arom_., *J* = 8.1 Hz); 7.63 (d, 1H, CH_arom_., *J* = 7.8 Hz); 9.01 (s, 1H, NH); 10.79 (s, 1H, NH). ^13^C-NMR (DMSO-*d*_6_) δ (ppm): 13.8 (CH_3_), 17.6 (CH_2_), 21.8 (CH_2_), 24.8 (CH_3_), 33.0 (CH_2_), 34.3 (CH_2_), 40.3 (CH_2_), 111.3 (C), 111.6 (CH), 118.1 (CH), 118.5 (CH), 120.9 (CH, CH), 122.6 (C), 126.2 (C), 127.3 (CH), 127.6 (CH), 130.4 (C), 134.5 (C), 136.2 (C), 140.6, 180.8 (C). HRMS (ESI) calc. for C_22_H_27_N_3_S [M − H]^−^: 364.5348, found: 364.5360.

*1-(2-(1H-Indol-3-yl)ethyl)-3-(3-chloro-4-fluorophenyl)thiourea* (**28**). Yield 91%. m.p. 162–164 °C. ^1^H-NMR (DMSO-*d*_6_) δ (ppm): 2.98 (t, 2H, CH_2_, *J* = 7.2 Hz); 3.76 (q, 2H, CH_2_, *J* = 5.7 Hz); 6.98 (t, 1H, CH_arom_., *J* = 8.1 Hz); 7.08 (t, 1H,CH_arom_., *J* = 7.2 Hz); 7.18 (d, 1H, CH_arom_., *J* = 2.1 Hz); 7.24–7.30 (m, 1H, CH_arom_.); 7.34 (d, 2H,CH_arom_., *J* = 8.7 Hz); 7.62 (d, 1H,CH_arom_., *J* = 7.8 Hz); 7.72 (d, 1H, CH_arom_., *J* = 6.6 Hz); 7.87 (bs, 1H, NH); 9.60 (s, 1H, NH); 10.84 (s, 1H, NH). ^13^C-NMR (DMSO-*d*_6_) δ (ppm): 24.4 (CH_2_), 44.5 (CH_2_), 111.4 (C), 111.5 (CH), 116.6 (d, *J* = 22 Hz, CH), 118.2 (CH), 118.4 (d, *J* = 18.6 Hz, CH), 121.0 (CH), 122.8 (CH), 123.6 (CH), 124.9 (d, *J* = 7.2 Hz, C), 127.2 (C), 136.3 (C), 136.5 (d, *J* = 3.3 Hz, C), 152.2 (C), 155.5 (d, *J* = 242.6 Hz, CH), 180.4 (C). HRMS (ESI) calc. for C_17_H_15_ClFN_3_S [M − H]^−^: 346.8375, found: 346.8378. *Crystal data:* crystal system triclinic, space group *P-1*, unit cell dimensions a = 8.592(2) Å, b = 12.4914(3) Å, c = 15.309(3) Å, α = 85.32(2)°, β = 81.17(2)°, γ = 88.87(2)°, V = 1678.1(6) Å^3^; Z = 4, d_c_ = 1.428 g/cm^3^, μ = 0.377 mm^−1^, *F*(000) = 720. A crystal of dimensions 0.40 × 0.20 × 0.03 mm was used for intensity measurements. Within the θ range 2.04–28.54° [−11 ≤ *h* ≤ 11, −16 ≤ *k* ≤ 16, −20 ≤ *l* ≤ 20], reflections collected 22058. The 5859 unique reflections [*R*(int) = 0.0360] were used for the refinement of 427 parameters. Final *R* indices on *F*^2^ for 5698 observed reflections [*I* > 2σ(*I*)] were: *R*_1_ = 0.0483, *wR*_2_ = 0.1019, goodness-of-fit 1.018, Δρ_max/min_ = 0.76/−0.80 e Å^−3^.

### 3.3. Biology

#### 3.3.1. In Vitro Evaluation of Antimicrobial Activity

The antibacterial activity of compounds was tested against a series of Gram-positive bacteria: *Staphylococcus aureus* ATCC 4163, *Staphylococcus aureus* ATCC 25923, *Staphylococcus aureus* ATCC 29213, *Staphylococcus aureus* ATCC 6538,*Staphylococcus epidermidis* ATCC 12228, *Bacillus subtilis* ATCC 6633, *Bacillus cereus* ATCC 11778, *Enterococcus hirae* ATCC 10541, *Micrococcus luteus* ATCC 9341, *Micrococcus luteus* ATCC 10240 and Gram-negative rods: *Escherichia coli* ATCC 10538, *Escherichia coli* ATCC 25922, *Escherichia coli* NCTC 8196, *Proteus vulgaris* NCTC 4635, *Pseudomonas aeruginosa* ATCC 15442, *Pseudomonas aeruginosa* NCTC 6749, *Pseudomonas aeruginosa* ATCC 27853, *Bordetella bronchiseptica* ATCC 4617. Antifungal activity was tested against yeasts: *Candida albicans* ATCC 10231, *Candida albicans* ATCC 90028, *Candida parapsilosis* ATCC 220191. Microorganisms used in this study were obtained from the collection of the Department of Pharmaceutical Microbiology, Medical University of Warsaw, Poland.

#### 3.3.2. Media, Growth Conditions and Antimicrobial Activity Assays

Antimicrobial activity was examined by the disc diffusion and Minimal Inhibitory Concentration (MIC) method under standard conditions, using Mueller–Hinton II agar medium (Becton Dickinson, Franklin Lakes, NJ, USA) for bacteria and RPMI agar with 2% glucose (Sigma, St. Louis, MO, USA) for yeasts, according to CLSI (previously NCCLS) guidelines [31]. Solutions containing the tested agents were prepared in methanol or DMSO. For the disc diffusion method, sterile paper discs (9 mm diameter, Whatman No. 3 chromatography filter paper) were dripped with the compound solutions tested to obtain 400 μg of substance per disc. Dry discs were placed on the surface of an appropriate agar medium. The results (diameter of the growth inhibition zone) were read after 18 h of incubation at 35 °C. MIC’s were examined by the twofold serial agar dilution technique [32]. Concentrations of the tested compounds in solid medium ranged from 3.125 to 400 μg/mL. The final inoculum of studied organisms was 10^4^ CFU/mL (colony forming units per mL), except the final inoculum for *E. hirae* ATCC 10541, which was 10^5^ CFU/mL. Minimal inhibitory concentrations were read off after 18 h of incubation at 35 °C.

#### 3.3.3. Inhibitory Activities against DNA Topoisomerase IV and DNA Gyrase 

*S. aureus* topoisomerase IV decatenation assay was performed with 200 ng of kinetoplast DNA (Inspiralis Limited, Norwich, UK) as a substrate. *S. aureus* DNA gyrase supercoiling assay was performed with 0.5 µg of relaxed pBR322 DNA (Inspiralis) as a substrate. Enzymes activity was detected by incubation for 30 min at 37 °C in a total reaction volume of 30 µL and in the presence of different concentrations of tested compounds and the reference ciprofloxacin at a concentration of 32 µg/mL. The reactions were terminated by adding an equal volume of STEB buffer (40% sucrose, 100 mM Tris-HCl pH 8, 1 mM EDTA, 0.5 mg/mL bromophenol blue), followed by extraction with 1 volume of chloroform/isoamyl alcohol (24:1). Then, 20 µL of the aqueous phase of each sample was loaded onto a 1% agarose gel. Following electrophoresis, gels were stained with ethidium bromide, visualized under UV light in a transilluminator (ChemiDoc MP, BioRad, Hercules, CA, USA) and analyzed by the Image Lab 6.0 software (Image Lab’s, Cleveland, OH, USA).

#### 3.3.4. Cells and Viruses

Cell lines were purchased from American Type Culture Collection (ATCC, Manassas, VA, USA). Cell lines supporting the multiplication of RNA and DNA viruses were the following: CD4+ human T-cells containing an integrated HTLV-1 genome (MT-4); Madin Darby Bovine Kidney (MDBK) [ATCC CCL 22 (NBL-1) *Bos taurus*]; baby hamster kidney (BHK-21) [ATCC CCL 10 (C-13) *Mesocricetus auratus*] and monkey kidney (Vero-76) [ATCC CRL 1587 *Cercopithecus aethiops*]. Human Immunodeficiency Virus type-1 (HIV-1) IIIB laboratory strain was obtained from the supernatant of the persistently infected H9/IIIB cells (NIH 1983). Viruses representative of ssRNA+ were: (i) *Flaviviridae*: yellow fever virus (YFV) [strain 17-D vaccine (Stamaril Pasteur J07B01)], bovine viral diarrhoea virus (BVDV) [strain NADL (ATCC VR-534)]; (ii) *Picornaviridae*: enterovirus B [coxsackievirus B5 (CV-B5), strain Ohio-1 (ATCC VR-29)], and enterovirus C [poliovirus type-1 (Sb-1), Sabin strain Chat (ATCC VR-1562)]. Viruses representative of ssRNA- were: (iii) *Pneumoviridae*: human respiratory syncytial virus (RSV) [strain A2 (ATCC VR-1540)]; (iv) *Rhabdoviridae*: vesicular stomatitis virus (VSV) [lab strain Indiana (ATCC VR 1540)]. The virus representative of dsRNA was: (v) Reoviridae: reovirus type-1 (Reo-1) [simian virus 12, strain 3651 (ATCC VR-214)]. DNA virus representatives were: v) *Poxviridae*: vaccinia virus (VV) [vaccine strain Elstree-Lister (ATCC VR-1549)]; (vi) *Herpesviridae*: human herpes 1 (HSV-1) [strain KOS (ATCC VR-1493)]. Mutants carrying NNRTI mutations used: Y181C mutant (NIH-N119) of HIV-1 derives from an AZT-sensitive clinical isolate passaged initially in CEM and then in MT-4 cells, in the presence of nevirapine (up to 10 µM); K103N + Y181C mutant (NIH A17) derives from an III_B_ strain passaged in H9 cells in the presence of BI-RG 587 (up to 1 µM); K103R + V179D + P225H mutant (EFV^R^) derives from an III_B_ strain passaged in MT-4 cells in the presence of efavirenz (up to 2 µM). N119, A17 and EFV^R^ stock solutions had titres of 1.2 × 10^8^, 2.1 × 10^7^, and 4.0 × 10^7^ CCID_50_/mL, respectively. Mutants carrying NRTI mutations used: AZT^R^ strain (67N, 70R, 215F, 219Q); MDR strain (74V, 41L, 106A, 215Y).

#### 3.3.5. Cytotoxicity Assays

Exponentially growing MT-4 cells were seeded at an initial density of 4 × 10^5^ cells/mL in 96-well plates in RPMI-1640 medium, supplemented with 10% fetal bovine serum (FBS), 100 units/mLpenicillin G and 100 μg/mL streptomycin. MDBK and BHK cells were seeded in 96-well plates at an initial density of 6 × 10^5^ and 1 × 10^6^ cells/mL, respectively, in minimum essential medium with Earle’s salts (MEM-E), l-glutamine, 1 mM sodium pyruvate and 25 mg/L kanamycin, supplemented with 10% horse serum (MDBK) or 10% foetal bovine serum (FBS) (BHK). Vero-76 cells were seeded in 96-well plates at an initial density of 4 × 10^5^ cells/mL, in Dulbecco’s Modified Eagle Medium (D-MEM) with l-glutamine and 25 mg/L kanamycin, supplemented with 10% FBS. Cell cultures were then incubated at 37 °C in a humidified, 5% CO_2_ atmosphere, in the absence or presence of serial dilutions of test compounds. Cell viability was determined after 48–96 h at 37 °C by MTT method for MT-4, Vero-76, MDBK and BHK [47].

Cell lines derived from human haematological tumours [CD4+ human acute T-lymphoblastic leukaemia (CCRF-CEM), human splenic B-lymphoblastoid cells (WIL-2NS), human acute B-lymphoblastic leukaemia (CCRF-SB)] were seeded at an initial density of 1 × 10^5^ cells/mL in 96 well plates in RPMI-1640 medium supplemented with 10% foetal calf serum (FCS), 100 units/mL penicillin G and 100 μg/mL streptomycin. Cell viability was determined after 96 h at 37 °C by the MTT method. All cell cultures were then incubated at 37 °C in a humidified, 5% CO_2_ atmosphere, in the absence or presence of serial dilutions of test compounds in culture medium. Before dilutions, compounds were dissolved in DMSO at 100 mM.

#### 3.3.6. Antiviral Assays

Activity against HIV-1 wt and mutant strains (N119, A17, EFV^R^, AZT^R^, MDR) was based on inhibition of virus-induced cytopathogenicity in exponentially growing MT-4 cell acutely infected with a multiplicity of infection (m.o.i.) of 0.01. Briefly, 50 μL of RPMI containing 1 × 10^4^ MT-4 cells were added to each well of flat-bottom microtitre trays, containing 50 μL of RPMI without or with serial dilutions of extracts or fractions. Then, 20 μL of a HIV-1 suspension containing 100 CCID_50_ were added. After 4-days incubation at 37 °C, cell viability was determined by the MTT method.

Compound’s activity against YFV and Reo-1 was based on inhibition of virus-induced cytopathogenicity in BHK-21 cells acutely infected with a m.o.i. of 0.01. Compound’s activity against BVDV was based on inhibition of virus-induced cytopathogenicity in MDBK cells acutely infected at a m.o.i. of 0.01. After a 3 or 4-days incubation at 37 °C, cell viability was determined by the MTT method, as described earlier [47]. Compound’s activity against CV-B5, Sb-1, VSV, VV, HSV-1 and RSV was determined by plaque reduction assays in infected Vero-76 cell monolayers, as described earlier [48,49]. Concentrations resulting in 50% inhibition (CC_50_ or EC_50_) were determined by linear regression analysis. Efavirenz, 2′-C-methylguanosine, 2′-C-methylcytidine, 6-azauridine, mycophenolic acid and acycloguanosine were used as reference inhibitors.

## Figures and Tables

**Table 1:**
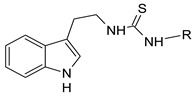
Structure and molar weight of the investigated compounds 1–28.

Number	R	Mol. Weight (g/mol)
**1**	Phenyl	295.40
**2**	4-Methoxyphenyl	325.43
**3**	4-Methylphenyl	309.43
**4**	4-Chlorophenyl	329.85
**5**	Benzoyl	323.41
**6**	3,4-Dichlorophenyl	364.29
**7**	3-Bromophenyl	374.30
**8**	4-Bromophenyl	374.30
**9**	Cyclohexyl	301.45
**10**	Benzyl	309.43
**11**	2-Fluorophenyl	313.39
**12**	3-Fluorophenyl	313.39
**13**	2-Chlorophenyl	329.85
**14**	3-Chlorophenyl	329.85
**15**	2-Bromophenyl	374.30
**16**	Phenethyl	323.46
**17**	4-Fluorophenyl	313.39
**18**	Allyl	259.37
**19**	Methallyl	273.40
**20**	Ethoxycarbonyl	291.37
**21**	4-Iodophenyl	421.30
**22**	Ethyl	247.36
**23**	3-Chloro-4-methylphenyl	343.87
**24**	3-Trifluoromethylphenyl	363.40
**25**	Methyl	233.33
**26**	3-Chloro-6-methylphenyl	343.87
**27**	4-Butyl-2-methylphenyl	365.53
**28**	3-Chloro-4-fluorophenyl	347.84

**Table 2:** Activities against Gram-positive bacteria—minimal inhibitory concentrations (MIC, µg/mL) and diameter of growth inhibitory zone (GIZ, mm, applied 400 µg per disc)*.*

Strains	6	14	15	19	Ref. ^1^
*S. aureus* NCTC 4163	6.25 (10)	50 (13)	50 (11)	400 (12)	0.25 (26)
*S. aureus* ATCC 25923	6.25 (15)	50 (14)	100 (11)	400 (12)	0.5 (26)
*S. aureus* ATCC 6538	12.5 (16)	50 (13)	50 (12)	200 (11)	0.25 (28)
*S. aureus* ATCC 29213	6.25 (15)	50 (15)	50 (11)	-	0.5 (22)
*S. epidermidis* ATCC 12228	12.5 (16)	50 (12)	-	-	0.25 (30)
*B. subtilis ATCC 6633*	12.5 (15)	50 (12)	50 (15)	400 (13)	<0.12 (38)
*B. cereus* ATCC 11778	6.25 (15)	25 (15)	50 (14)	200 (13)	0.25 (26)
*M. luteus* ATCC 9341	12.5 (15)	200 (12)	200 (13)	200 (13)	0.25 (26)
*M. luteus* ATCC 10240	6.25 (23)	12.5 (20)	50 (14)	50 (14)	1 (25)

^1^ Ref.—ciprofloxacin (GIZ—5 µg/9 mm disc). Lack of the growth inhibition area.

**Table 3:** Activity of compounds against hospital methicillin-susceptible strains of *S. aureus* (MSSA), methicillin-resistant strains of *S. aureus* (MRSA) and *S. epidermidis* (MRSE)—minimal inhibitory concentrations (MIC, µg/mL).

***S. aureus* MSSA**	**440/11**	**441/11**	**442/11**	**443/11**	**444/11**	**445/11**	**446/11**	**447/11**	**448/11**	**449/11**
**6**	6.25	6.25	6.25	6.25	6.25	6.25	6.25	6.25	6.25	6.25
**14**	25	25	25	25	12,5	25	25	25	25	25
Ciprofloxacin	0.4	0.4	0.4	0.4	0.8	0.4	0.4	0.8	0.8	0.4
***S. aureus* MRSA**	**389/10**	**390/10**	**391/10**	**392/10**	**393/10**	**394/10**	**399/10**	**450/11**	**451/11**	**452/11**
**6**	6.25	6.25	6.25	6.25	6.25	6.25	6.25	6.25	6.25	6.25
**14**	25	25	25	25	25	25	25	25	25	25
Ciprofloxacin	25	100	100	100	50	25	200	100	100	200
***S. epidermidis* MRSE**	**430/11**	**431/11**	**432/11**	**433/11**	**434/11**	**435/11**	**436/11**	**437/11**	**438/11**	**439/11**
**6**	6.25	6.25	6.25	6.25	12.5	6.25	6.25	6.25	6.25	6.25
**14**	25	6.25	25	25	25	25	50	25	50	25
**15**	400	50	50	50	50	100	100	100	200	50
**19**	12.5	100	12.5	50	12.5	50	100	200	100	50
Ciprofloxacin	0.8	6.25	50	50	50	0.4	0.4	0.8	0.4	50

**Table 4:** Inhibitory activities against DNA topoisomerase IV and DNA gyrase, expressed as IC_50_ ± SEM (µg/mL).

Compounds	*S. aureus* Topoisomerase IV	*S. aureus* DNA Gyrase
**6**	28.90 ± 0.30	72.60 ± 1.2
Ciprofloxacin	1.70 ± 0.15	3.55 ± 0.13

IC_50_—half of the maximal inhibitory concentration.

**Table 5:** Antiviral activity of thiourea derivatives against HIV-1 and ssRNA+ (BVDV, YFV, CV-B5, Sb-1) viruses and cytotoxicity against the cell lines used in the assays.

Compounds	MT-4 ^a^ CC_50_	HIV-1 ^b^ EC_50_	MDBK ^c^ CC_50_	BVDV ^d^ EC_50_	BHK ^e^ CC_50_	YFV ^f^ EC_50_	Vero-76 ^g^ CC_50_	CV-B5 ^h^ EC_50_	Sb-1 ^i^ EC_50_
**1**	31	>31	>100	>100	48	>48	64	>64	>64
**2**	32	>32	>100	>100	44	>44	56	>56	>56
**3**	33	>33	>100	≥100	16	>16	75	>75	>75
**4**	45	>45	>100	>100	47	>47	55	>55	>55
**5**	38	>38	67	**20 ± 1.5**	34	>34	29	>29	>29
**6**	45	>45	27	>27	10	>10	13	>13	>13
**7**	45	>45	55	**20 ± 2.0**	10	>10	12	>12	>12
**8**	45	**8.7 ± 0.4**	52	>52	10	>10	13	>13	>13
**9**	49	>49	55	>55	13	>13	14	>14	>14
**10**	78	>78	>100	>100	52	>52	>100	**30 ± 2.4**	>100
**11**	48	>48	69	>69	34	>34	73	>73	>73
**12**	47	>47	49	>49	16	>16	60	>60	>60
**13**	47	>47	49	>49	11	>11	53	**12 ± 0.9**	>53
**14**	42	>42	32	>32	6.0	>6.0	32	**9.0 ± 1.2**	>32
**15**	46	>46	48	>48	28	>28	46	>46	>46
**16**	49	>49	>100	**46**	18	>18	>100	**18 ± 1.7**	>100
**17**	44	>44	53	>53	30	>30	53	>53	>53
**18**	>100	>100	>100	>100	>100	>100	>100	>100	>100
**19**	>100	>100	>100	≥100	35	>35	89	>89	>89
**20**	>100	>100	>100	**35 ± 2.8**	>100	>100	>100	>100	>100
**21**	**2.4 ± 0.2**	>2.4	>100	>100	11	>11	11	>11	>11
**22**	>100	>100	>100	>100	>100	>100	>100	>100	>100
**23**	44	>44	25	>25	10	>10	17	>17	>17
**24**	44	>44	23	>23	9.0	>9.0	16	>16	>16
**25**	>100	>100	>100	>100	>100	>100	>100	>100	>100
**26**	45	>45	42	**15 ± 1.2**	12	>12	17	>17	>17
**27**	45	>45	11	>11	9.5	>9.5	12	>12	>12
**28**	45	>45	37	**13 ± 1.2**	30	>30	32	**20 ± 2.1**	>32
Efavirenz	40	0.002 ± 0.0002							
2′-C-methyl-guanosine			>10	1.1 ± 0.1	>10	1.9 ± 0.1			
2′-C-methyl-cytidine							>100	18 ± 0.9	7.3 ± 0.4

Data represent mean values for three independent determinations. Standard deviations are reported for the more active compounds. Also for the others values the variation was less than 15%. ^a^ Compound concentration (µM) required to reduce the proliferation of mock-infected MT-4 cells by 50%. ^b^ Compound concentration (µM) required to achieve 50% protection of MT-4 cells from HIV-1 induced cytopathogenicity. ^c^ Compound concentration (µM) required to reduce the viability of mock-infected MDBK cells by 50%. ^d^ Compound concentration (µM) required to achieve 50% protection of MDBK cells from BVDV-induced cytopathogenicity. ^e^ Compound concentration (µM) required to reduce the viability of mock-infected BHK cells by 50%. ^f^ Compound concentration (µM) required to achieve 50% protection of BHK cells from YFV-induced cytopathogenicity. ^g^ Compound concentration (µM) required to reduce the viability of mock-infected VERO-76 cells by 50%. ^h,i^ Compound concentration (µM) required to reduce the plaque number of CV-B5 ^h^, Sb-1 ^i^ by 50% in VERO-76 monolayers.

**Table 6:** Antiviral activity of thiourea derivatives against ssRNA-(RSV, VSV), dsRNA (Reo-1) and dsDNA (VV, HSV-1) viruses and cytotoxicity against the cell lines used in the assays.

Compounds	BHK ^a^ CC_50_	Reo-1 ^b^ EC_50_	Vero-76 ^c^ CC_50_	RSV ^d^ EC_50_	VSV ^e^ EC_50_	VV ^f^ EC_50_	HSV-1 ^g^ EC_50_
**1**	48	>48	64	>64	>64	>64	>64
**2**	44	>44	56	>56	>56	>56	>56
**3**	16	>16	75	**20 ± 3.0**	>75	>75	>75
**4**	47	>47	55	>55	>55	>55	>55
**5**	34	>34	29	>29	>29	>29	>29
**6**	10	>10	13	>13	>13	>13	>13
**7**	10	>10	12	>12	>12	>12	>12
**8**	10	>10	13	>13	>13	>13	>13
**9**	13	>13	14	>14	>14	>14	>14
**10**	52	>52	>100	>100	>100	>100	>100
**11**	34	>34	73	>73	>73	>73	>73
**12**	16	>16	60	>60	>60	>60	>60
**13**	11	>11	53	>53	>53	>53	>53
**14**	6.0	>6.0	32	>32	>32	>32	>32
**15**	28	>28	46	**20 ± 2.5**	>46	>46	>46
**16**	18	>18	>100	**30 ± 2.6**	>100	>100	>100
**17**	30	>30	53	>53	>53	>53	>53
**18**	>100	**61**	>100	>100	>100	>100	>100
**19**	35	>35	89	>89	>89	>89	>89
**20**	>100	>100	>100	>100	>100	>100	>100
**21**	11	>11	11	>11	>11	>11	>11
**22**	>100	>100	>100	>100	>100	>100	>100
**23**	10	>10	17	>17	>17	>17	>17
**24**	9.0	>9.0	16	>16	>16	>16	>16
**25**	>100	>100	>100	>100	>100	>100	>100
**26**	12	>12	17	>17	>17	>17	>17
**27**	9.5	>9.5	12	>12	>12	>12	>12
**28**	30	>30	32	>32	>32	>32	>32
2′-C-methyl-cytidine	>100	10 ± 1.2					
6-Aza-uridine			12	1.4 ± 0.06			
Mycophenolic acid			13			1.5 ± 0.2	
Acycloguanosine			>100				3.0 ± 0.2

Data represent mean values for three independent determinations. Standard deviations are reported for the more active compounds. Also for the others values the variation was less than 15%. ^a^ Compound concentration (µM) required to reduce the proliferation of mock-infected BHK cells by 50%. ^b^ Compound concentration (µM) required to achieve 50% protection of BHK cells from Reo-1-induced cytopathogenicity. ^c^ Compound concentration (µM) required to reduce the viability of mock-infected VERO-76 cells by 50%. ^d–g^ Compound concentration (µM) required to reduce the plaque number of RSV ^d^, VSV ^e^, VV ^f^, HSV-1 ^g^ by 50% in VERO-76 monolayers.

**Table 7:** Cytotoxicity and antiviral activity of tryptamine derivative **8** against HIV-1 and its NRTI- and NNRTI-resistant mutants*.*

Compounds	CC_50_^a^	EC_50_^b^					
	MT	HIV-1	N119	A17	EFV^R^	AZT^R^	MDR
**8**	45	**8.7 ± 0.8**	>45	**12 ± 1.5**	>45	**9.0 ± 1.0**	**9.0 ± 0.8**
Efavirenz	40	0.002 ± 0.0003	0.018 ± 0.002	0.075 ± 0.009	7.0 ± 0.8	0.001 ± 0.0002	NA
AZT	45	0.022 ± 0.004	0.025 ± 0.004	0.01 ± 0.001	0.02 ± 0.001	0.35 ± 0.04	0.08 ± 0.006
Nevirapine	>100	0.080 ± 0.005	6.3 ± 0.05	80	100	0.078 ± 0.008	5.0 ± 0.6

Data represent mean values for three independent determinations. ^a^ Compound concentration (µM) required to reduce the proliferation of mock-infected MT-4 cells by 50%. ^b^ Compound concentration (µM) required to achieve 50% protection of MT-4 cells from HIV-1 induced cytopathogenicity.

**Table 8:** Cytotoxicity of tryptamine derivative **21** against human leukaemia/lymphoma cell lines.

Compounds	CC_50_^a^		
	CCRF-CEM ^b^	WIL-2NS ^c^	CCRF-SB ^d^
**21**	5.0 ± 0.8	5.8 ± 1.0	12 ± 1.6
Doxorubicin	0.02 ± 0.002	0.02 ± 0.003	0.03 ± 0.003

Data represent mean values for three independent determinations. ^a^ Compound concentration (µM) required to reduce cell proliferation by 50%, under conditions allowing untreated controls to undergo at least three consecutive rounds of multiplication. ^b^ CD4+ human acute T-lymphoblastic leukaemia. ^c^ Human splenic B-lymphoblastoid cells. ^d^ Human acute B-lymphoblastic leukemia.

## Supplementary Materials

The following are available online.
