# Supplementary material for: Synthesis and Biological Evaluation of Novel Indole-Derived Thioureas
Source: Molecules. 2018 Oct 7;23(10):2554. doi: 10.3390/molecules23102554 (PMC6222422; doi:10.3390/molecules23102554)

Supplementary material

Figure 1S. View of conformers observed in solid for molecules 8 (top) and 28 (bottom). Displacement ellipsoids drawn at 50% probability level.

8

28

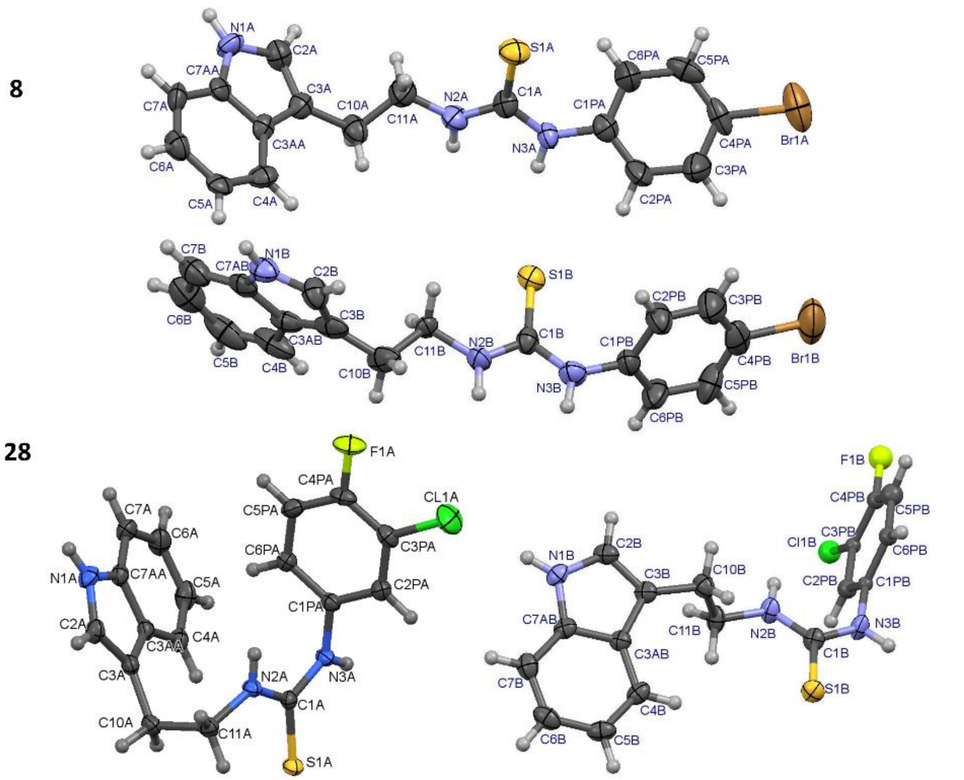

Figure 1S. View of conformers observed in solid for molecules 8 (top) and 28 (bottom). Displacement ellipsoids drawn at 50% probability level. Both compounds are conformationally unstable which is observed in the solid. The molecules **8** and **28** crystallize as two conformers differing in the orientation of the thiourea and indole groups, and in the patterns of noncovalent intermolecular contacts.

The ^{1}H NMR, ^{13}C NMR spectra of the selected compounds.

Spectrum 1. ^{1}H NMR of compd **6** (300 MHz, DMSO-d_6).
Spectrum 2. ^{13}C NMR of compd **6** (75.4 MHz, DMSO-d_6).
Spectrum 3. ^{1}H NMR of compd **8** (300 MHz, DMSO-d_6).
Spectrum 4. ^{13}C NMR of compd **8** (75.4 MHz, DMSO-d_6).
Spectrum 5. ^{1}H NMR of compd **14** (300 MHz, DMSO-d_6).
Spectrum 6. ^{13}C NMR of compd **14** (75.4 MHz, DMSO-d_6).
Spectrum 7. ^{1}H NMR of compd **15** (300 MHz, DMSO-d_6).
Spectrum 8. ^{13}C NMR of compd **15** (75.4 MHz, DMSO-d_6).
Spectrum 9. ^{1}H NMR of compd **19** (300 MHz, DMSO-d_6).
Spectrum 10. ^{13}C NMR of compd **19** (75.4 MHz, DMSO-d_6).

Spectrum 1. ^{1}H NMR of compound **6** (300 MHz, DMSO-d_6).

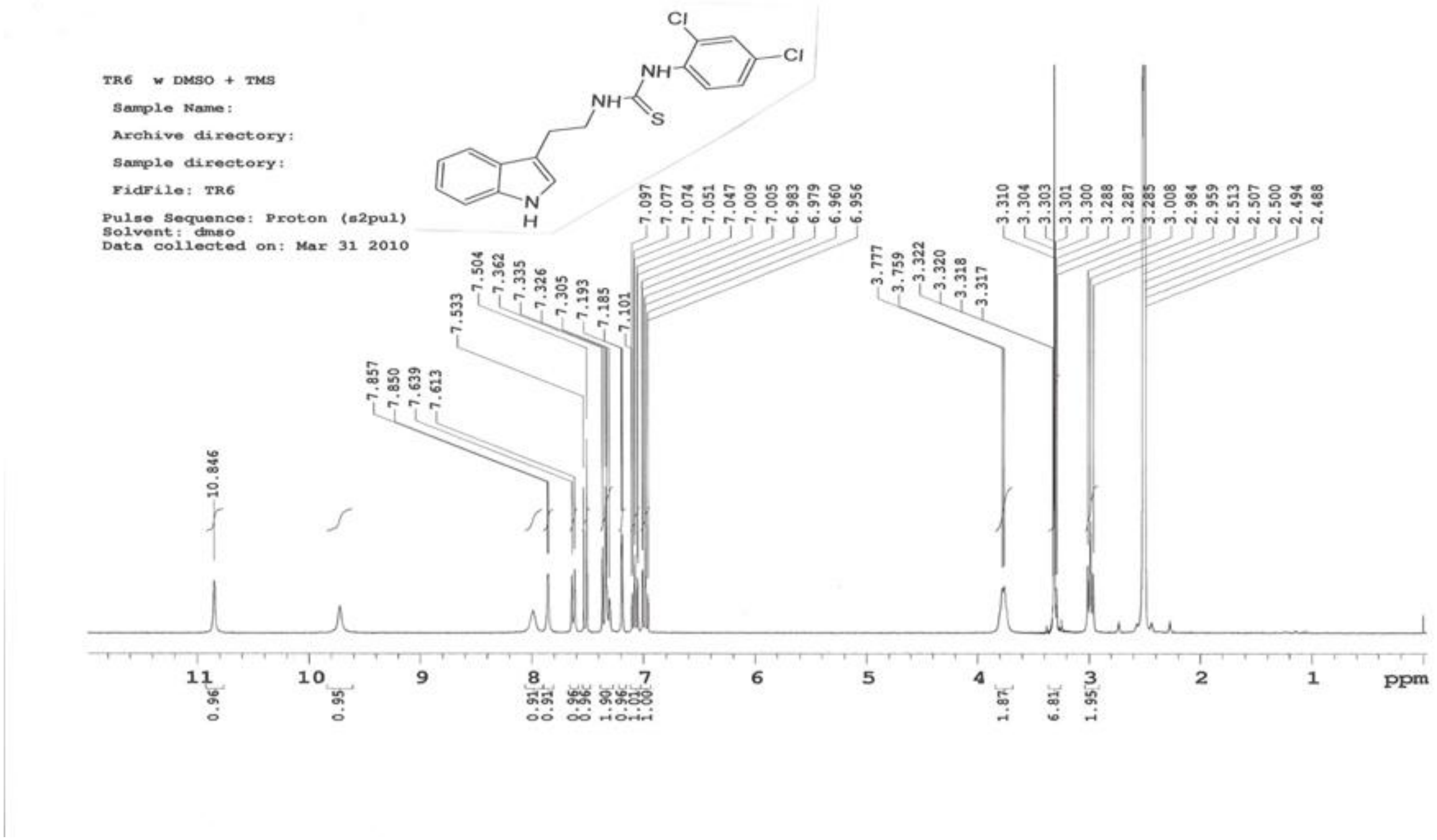

Spectrum 2. ^{13}C NMR of compound **6** (75.4 MHz, DMSO-d_6).

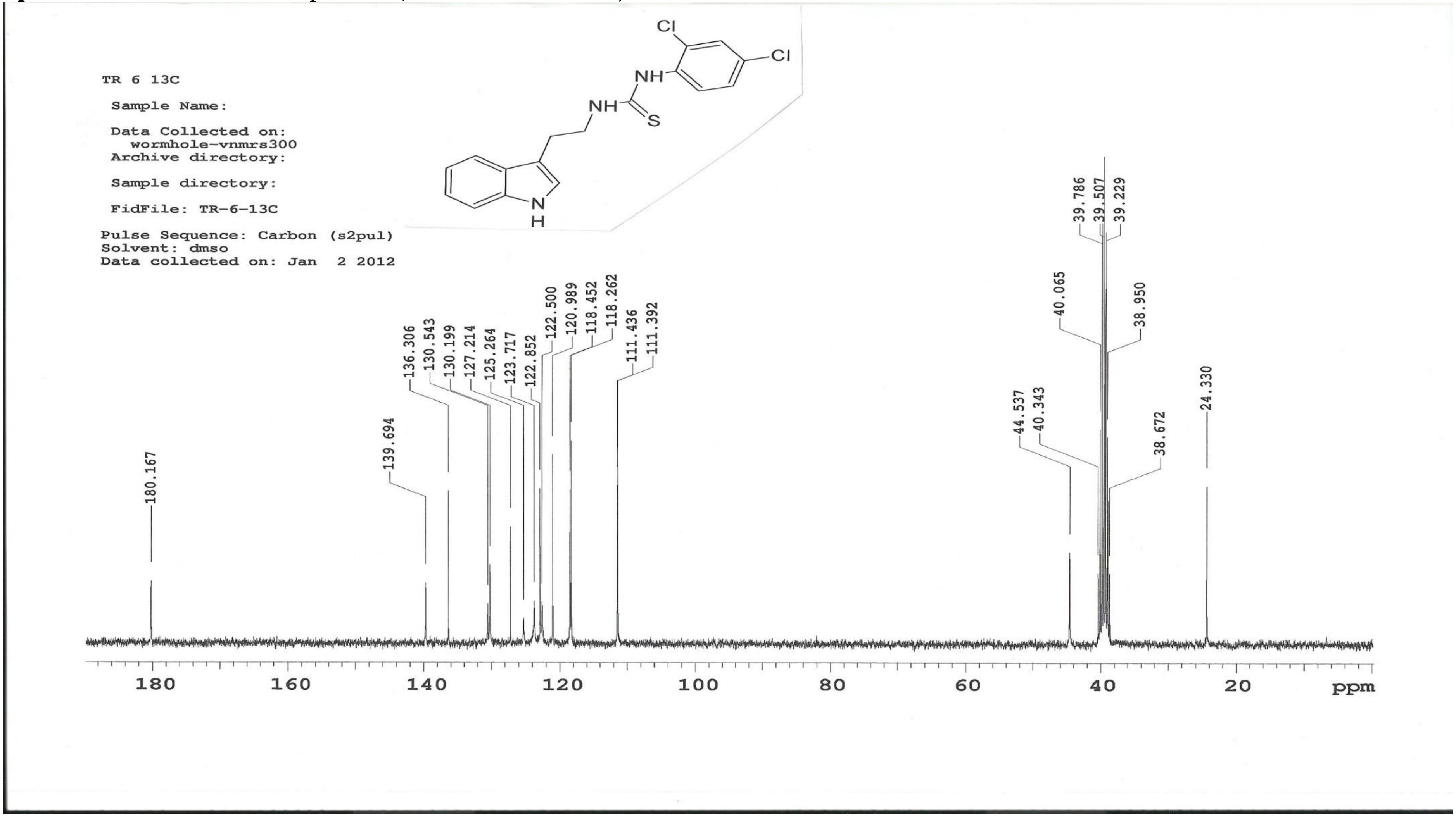

Spectrum 3. ^{1}H NMR of compound **8** (300 MHz, DMSO-d_6).

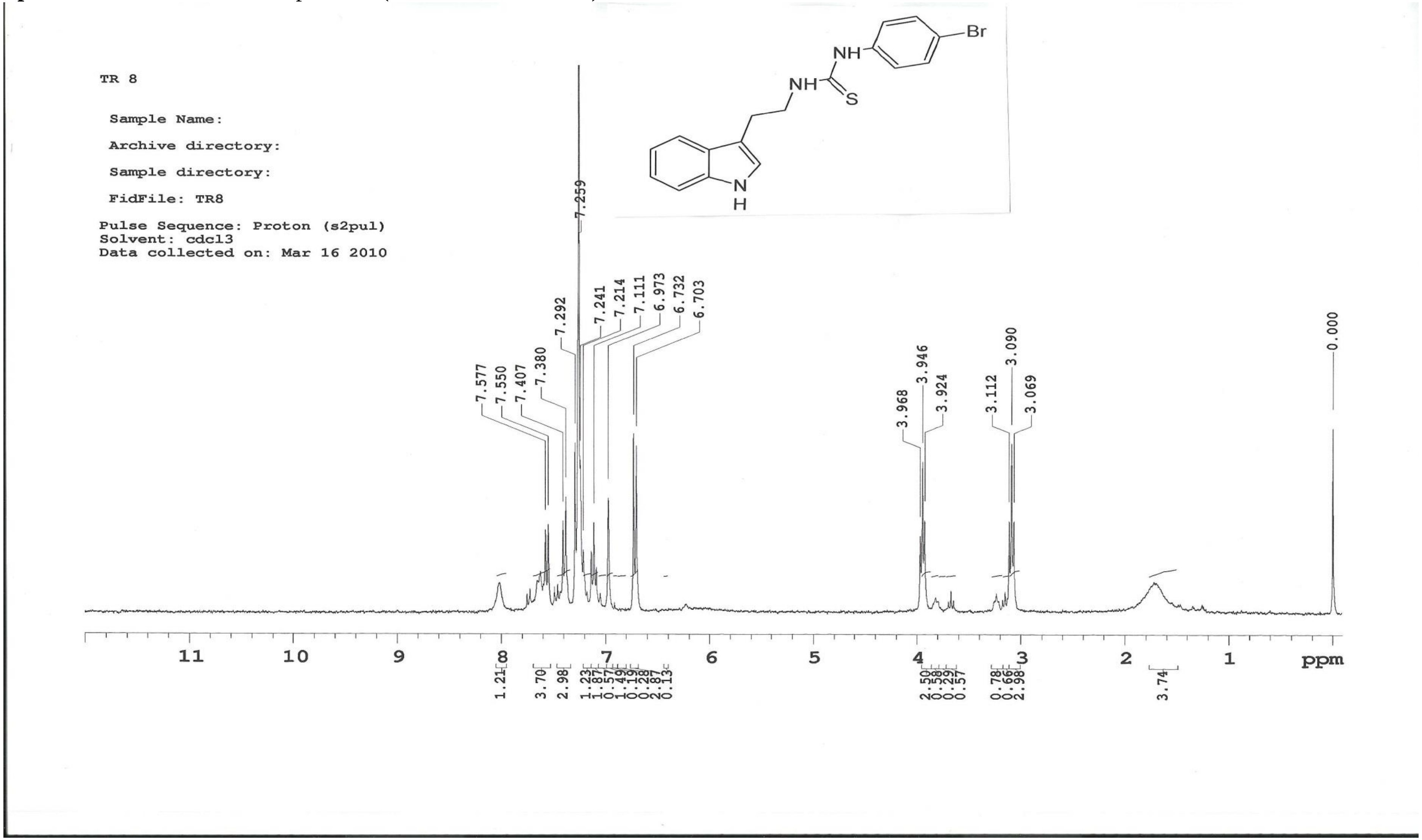

Spectrum 4. ^{13}C NMR of compound **8** (75.4 MHz, DMSO-d_6).

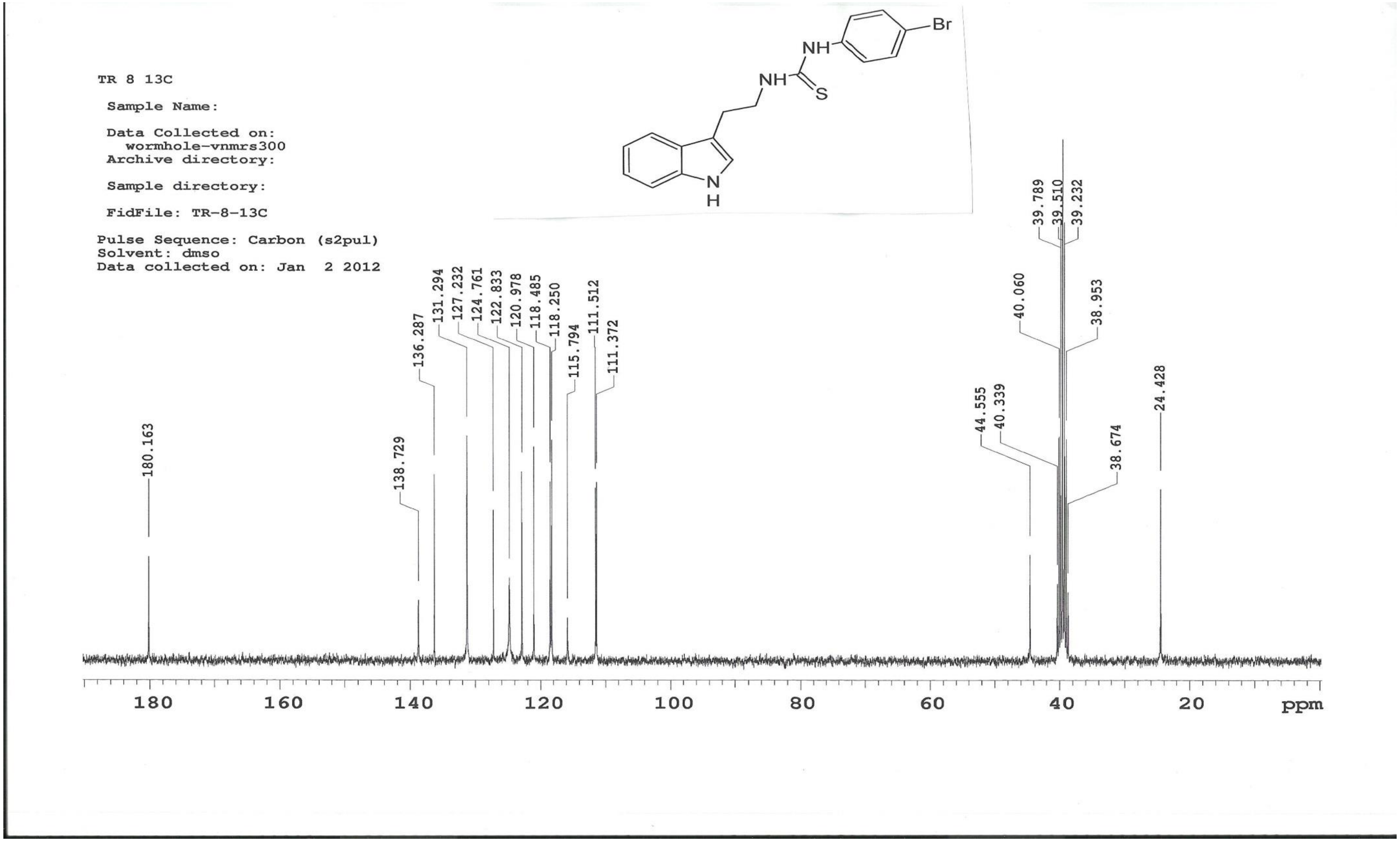

Spectrum 5. ^{1}H NMR of compound **14** (300 MHz, DMSO-d_6).

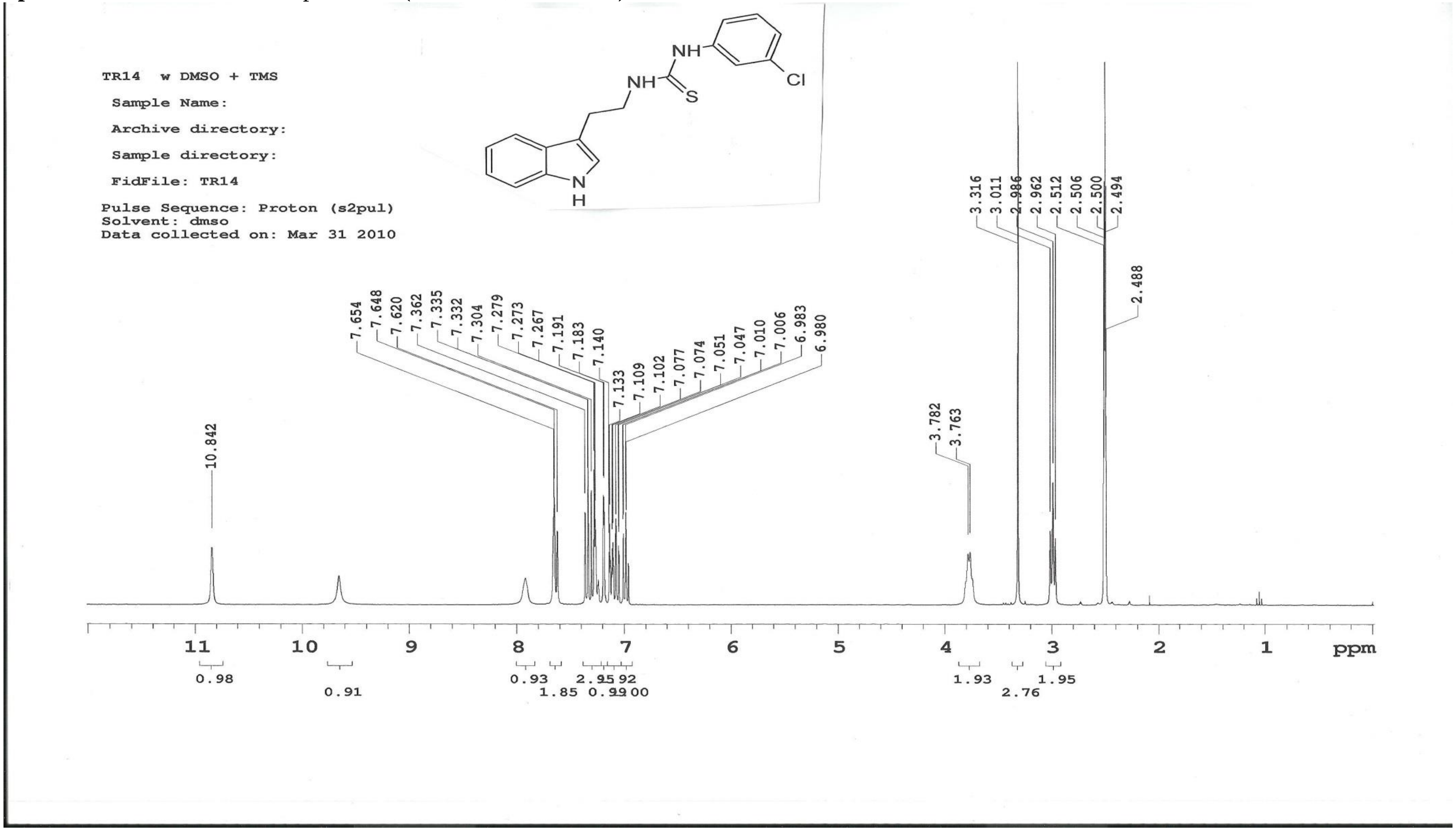

Spectrum 6. ^{13}C NMR of compound **14** (75.4 MHz, DMSO-d_6).

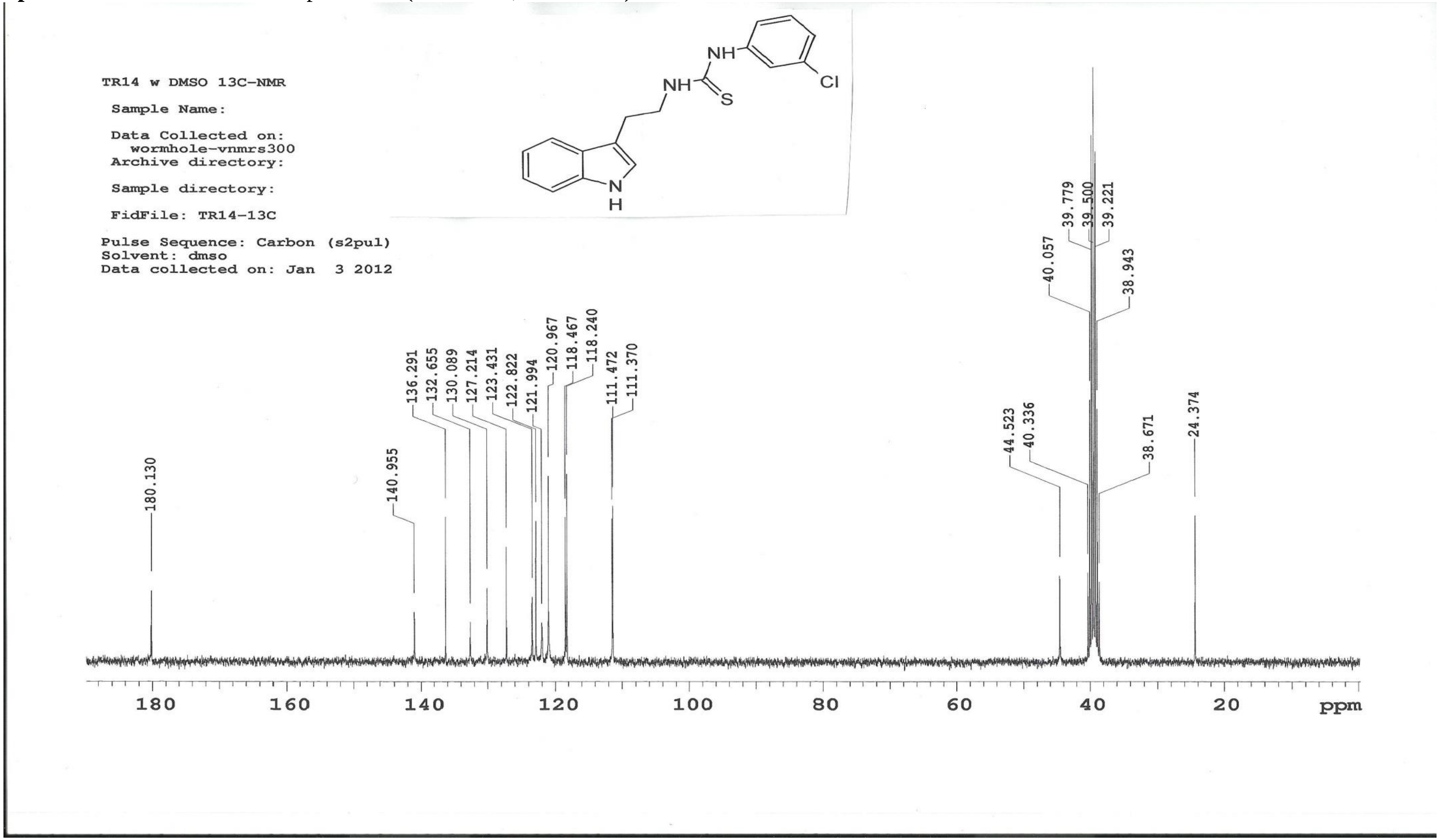

Spectrum 7. ^{1}H NMR of compound **15** (300 MHz, DMSO-d_6).

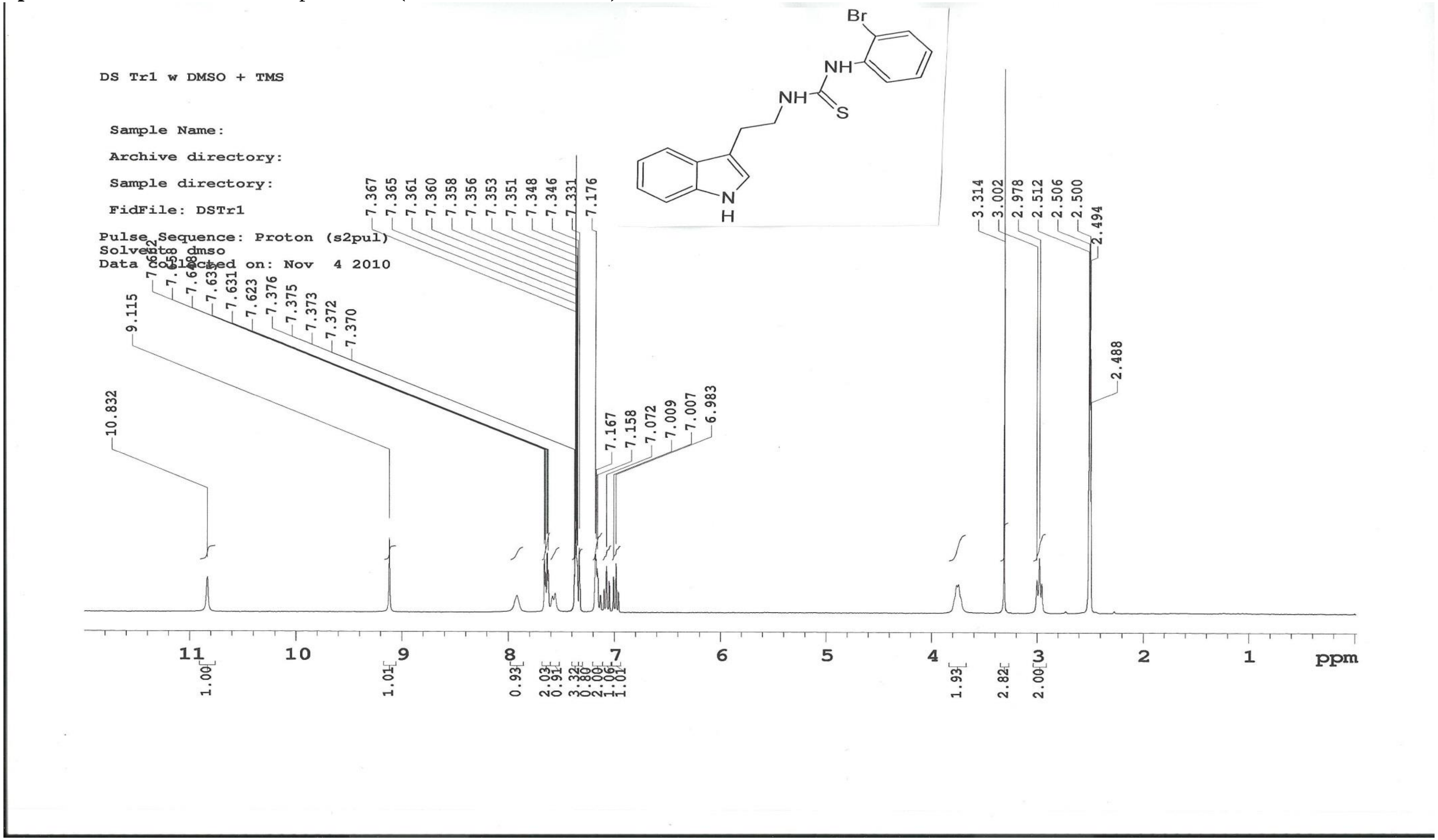

Spectrum 8. ^{13}C NMR of compound **15** (75.4 MHz, DMSO-d_6).

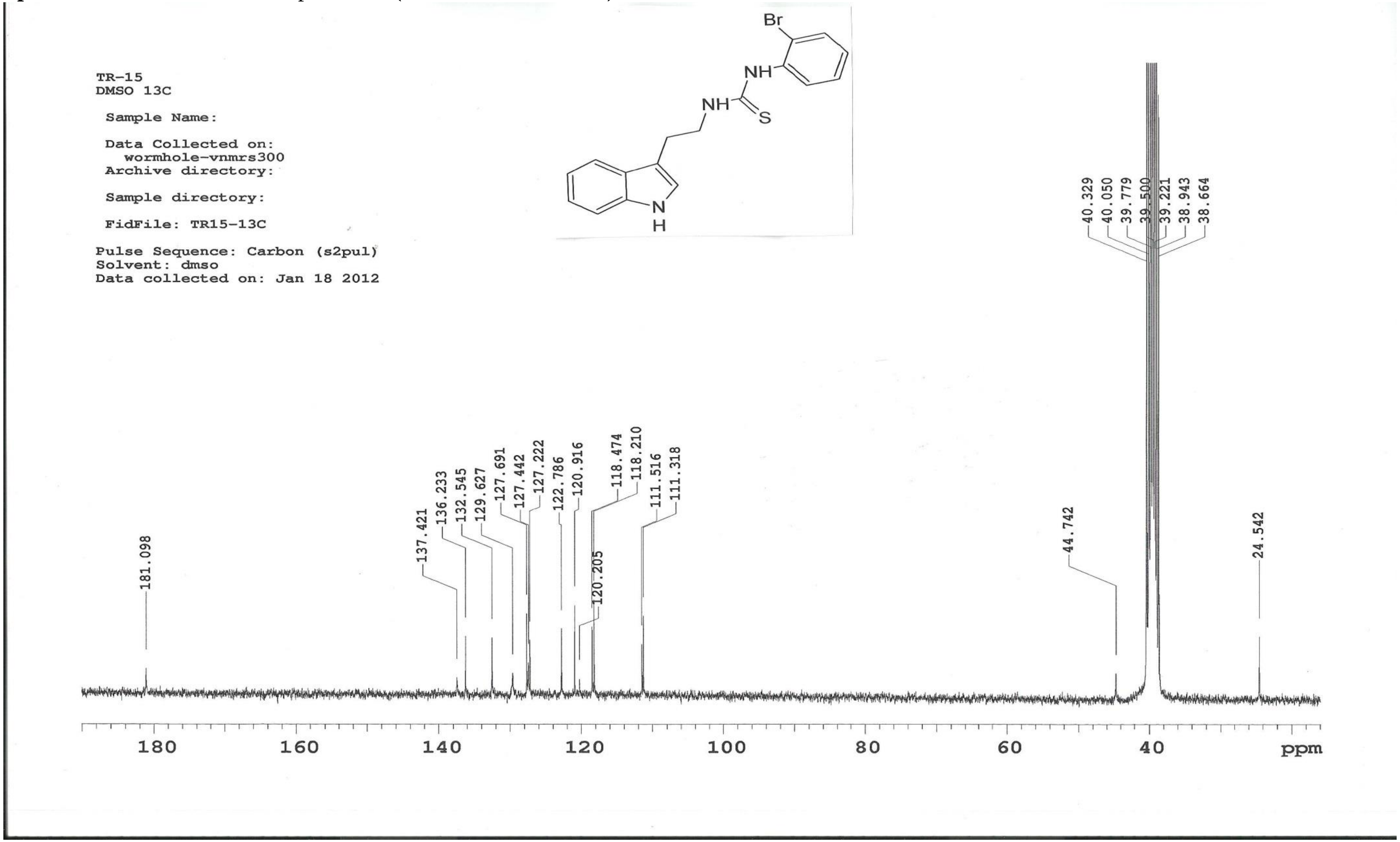

Spectrum 9. ^{1}H NMR of compound **19** (300 MHz, DMSO-d_6).

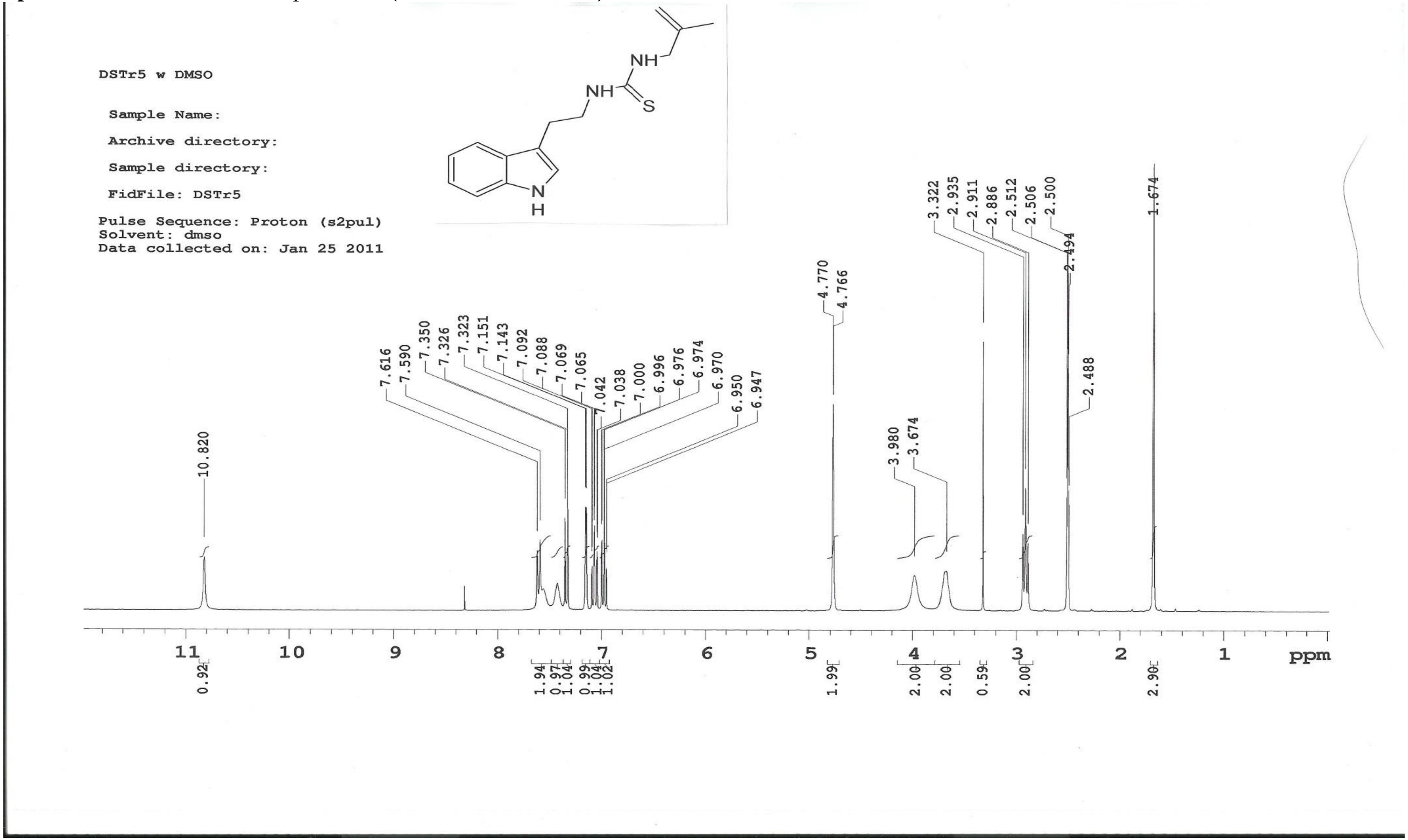

Spectrum 10. ^{13}C NMR of compound **19** (75.4 MHz, DMSO-d_6).

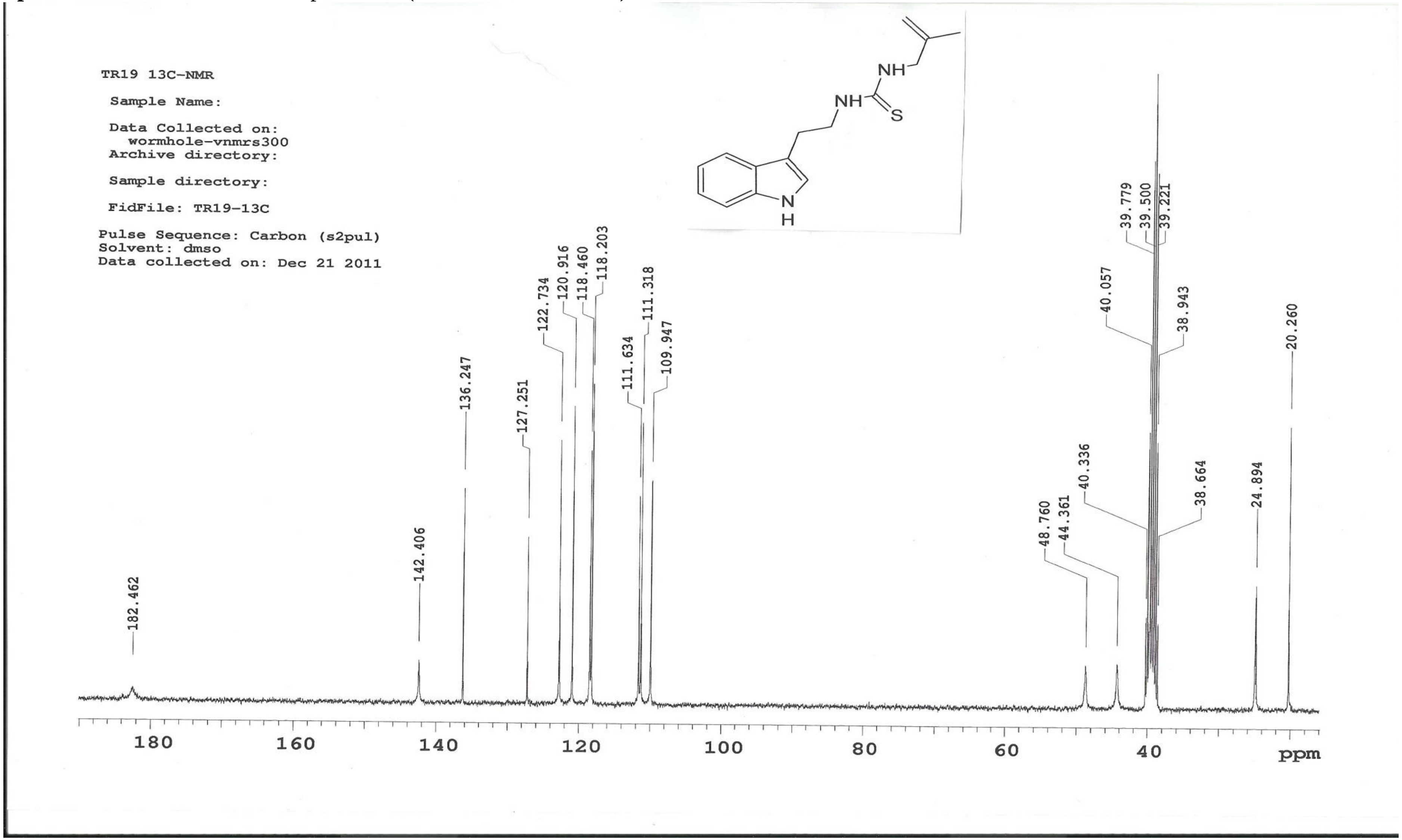

Supplement: Supplementary file 1 [file molecules-23-02554-s001.pdf]
